# Cranberry (*Vaccinium macrocarpon*) Juice Precipitate Pigmentation Is Mainly Polymeric Colors and Has Limited Impact on Soluble Anthocyanin Loss

**DOI:** 10.3390/antiox10111788

**Published:** 2021-11-09

**Authors:** Matthew R. Dorris, Bradley W. Bolling

**Affiliations:** 1 Department of Chemistry, University of Wisconsin-Madison, Madison, WI 53706, USA; mdorris@chem.wisc.edu; 2 Department of Food Science, University of Wisconsin-Madison, Madison, WI 53706, USA

**Keywords:** cranberry, anthocyanin, juice, polymeric color, proanthocyanidin, precipitate, cloud, haze, MALDI

## Abstract

Anthocyanins degrade in fruit juice during storage, reducing juice color quality and depleting the health-promoting components of juice. Common water-soluble products of anthocyanins’ chemical degradation are known, but little is known about the contribution of the insoluble phase to loss processes. Cranberry juice and isolated anthocyanins were incubated at 50 °C for up to 10 days to determine polyphenol profiles and degradation rates. Anthocyanin-proanthocyanidin heteropolymers were analyzed via Matrix Assisted Laser Desorption/Ionization (MALDI)- Time of Flight (TOF) Mass Spectrometry (MS). Formation of soluble protocatechuic acid accounted for 260 ± 10% and insoluble materials for 80 ± 20% of lost soluble cyanidin-glycosides in juice, over-representations plausibly due to quercetin and (epi)catechin in cranberry juice and not observed in the values of 70 ± 20% and 16 ± 6% in the purified anthocyanin system. Loss processes of soluble peonidin-glycosides were better accounted for, where 31 ± 2% were attributable to soluble vanillic acid formation and 3 ± 1% to insoluble materials in cranberry juice and 35 ± 5% to vanillic acid formation and 1.6 ± 0.8% to insoluble materials in the purified anthocyanin system. Free anthocyanins were below quantifiable levels in precipitate, implying most anthocyanins in precipitate were polymeric colors (PCs). PCs in the precipitate included cyanidin- and peonidin-hexosides and -pentosides covalently bonded to procyanidins. Therefore, formation of cranberry juice precipitate does not deplete a large portion of soluble anthocyanins; rather, the precipitate’s pigmentation results from PCs that are also present in the soluble phase.

## 1. Introduction

Understanding anthocyanin content of fruit juices and how anthocyanins change over juice shelf life is important because anthocyanins define juice color quality, an important parameter for consumer appeal, and because anthocyanins are important to human health. In addition to the benefits of regular consumption of polyphenols including reduced risk of all-cause mortality, cardiovascular disease mortality [1], certain cancers [2], diabetes [3], and Parkinson disease [4], consumption of anthocyanin-rich juices specifically has shown benefit for chronic inflammation [5], cognitive function [6,7], and blood pressure [6] in human intervention studies. Notably, clinical and epidemiological studies imply that increased anthocyanin and polyphenol intake may be beneficial for reducing the risk of cognitive decline, neurodegenerative diseases, and cardiovascular diseases in part, due to the antioxidant properties of these compounds [8].

There has been debate among public health experts and dieticians about whether fruit juice should be recommended for consumption because of its higher sugar content and lower fiber content compared to whole fruit, though recent analysis implies that consumption of 100% fruit juice is an effective way to increase fruit consumption as part of a healthy diet [9]. Because the macronutrients of fruit juice are seemingly less beneficial for consumers than whole fruit, having a high content of health-promoting antioxidants is especially important for the quality of fruit juices. The polyphenols in fruit juice do not have equivalent antioxidant capacity [10,11], so knowing the composition of polyphenols in fruit juices and how polyphenol composition changes over juice shelf life is important to understanding the potential benefits of fruit juice consumption. Unfortunately, the dynamics of anthocyanin loss and development of polymeric color from anthocyanins are not fully understood [12,13,14,15].

Thermal treatment of anthocyanins in plant extracts and juices leads to loss of anthocyanins via scission of the C ring, resulting in the formation of phloroglucinaldehyde from the A ring and a hydroxybenzoic acid from the B ring [16,17,18,19,20,21]. The rates of degradation vary with the anthocyanin, temperature, light exposure, and matrix [22,23,24], and how well hydroxybenzoic acid formation correlates with anthocyanin loss varies heavily with analyte and aqueous extract or juice [13,16,17,18,19]. Consequently, the concentration of pertinent hydroxybenzoic acids such as gallic acid, protocatechuic acid, and vanillic acid is regularly quantitated alongside anthocyanins in analyses of juice and extracts [13,16,17,18,19,20,25,26]. Anthocyanins are also known to form pyranoanthocyanins and condensation products with proanthocyanidins, though these products tend to be larger sinks for anthocyanins in wine systems where alpha-dicarbonyls and aldehydes are formed during fermentation than in juice [22,27].

Precipitation has been suggested as a pertinent mechanism of loss for soluble anthocyanins [13,28,29,30]. Several researchers have characterized insoluble materials from juices, wines, and model systems. The composition varies greatly with the fruit and preparation process, but consists largely of protein, saccharides and in some systems lipids or tannins [28,29,31,32]. The extent to which precipitation contributes to anthocyanin loss processes in juice and similar beverages has not been adequately defined. In this study, we hypothesized that anthocyanin loss processes are not well accounted for and include not only established degradation to hydroxybenzoic acids but also precipitation; that anthocyanin loss processes differ between whole juice and a simplified system of cranberry juice anthocyanins in buffer because of the known contribution of juice matrix components [33,34,35,36,37]; and that non-specific spectrophotometric methods typically used for juice quality analysis do not accurately and completely capture anthocyanin loss processes.

## 2. Materials and Methods

### 2.1. Reagents

Methanol, acetone, and ethyl acetate (HPLC grade) for extraction and chromatography; methanol (Optima) for mass spectrometry sample preparation; potassium metabisulfite (FCC/NF grade); and potassium chloride (ACS grade) were obtained from Thermo Fisher Scientific (Waltham, MA, USA). Water was ultrapure grade, purified to at least 18.1 MΩ cm using a Barnstead water filtration system (Thermo Fisher Scientific). Hydrochloric acid (37 wt%, 99.999% trace metals basis); anhydrous, monobasic potassium phosphate (ACS reagent grade); anhydrous sodium acetate (reagent plus grade); polypropylene glycol P 2000 (PPG); and 2,5-dihydroxybenzoic acid (DHB) were obtained from Sigma-Aldrich (St. Louis, MO, USA). Formic acid (99% purity) and *tert*-butylhydroquinone (97% purity, TBHQ) were obtained from Acros Organics (Morris Plains, NJ, USA). Anhydrous ethanol (200 proof, USP specifications) was obtained from Decon labs (King of Prussia, PA, USA). Dimethyl sulfoxide (ACS grade, DMSO) was obtained from VWR International (Radnor, PA, USA). All solvents used for HPLC analysis were filtered through 0.20 µm nylon membrane filters obtained from MilliporeSigma (Burlington, MA, USA).

Gallic acid, monohydrate (≥99% purity) was obtained from Acros Organics. Cyanidin chloride (≥95% purity) and 3,4-dihydroxybenzoic acid (97% purity, PCA) were obtained from Sigma-Aldrich. Vanillic acid (98% purity, VA) was obtained from Alfa Aesar (Ward Hill, MA, USA). Cyanidin-3-*O*-galactoside (cy3gal) chloride and cyanidin-3-*O*-glucoside (cy3glu) chloride were of 94.0% purity and obtained from ChromaDex Standards (Longmont, CO, USA). Cyanidin-3-*O*-arabinoside (cy3ar) chloride, peonidin-3-*O*-galactoside (p3gal) chloride, peonidin-3-*O*-glucoside (p3glu) chloride, and peonidin-3-*O*-arabinoside (p3ar) chloride were of ≥95% purity and obtained from Extrasynthese (Genay, France). Peonidin chloride (≥ 98% purity) was obtained from Cayman Chemical Company (Ann Arbor, MI, USA). These eleven polyphenol standards were stored as stock solutions of 2–6 mM in methanol at −80 °C.

### 2.2. Juice Preparation and Accelerated Aging

Aliquots of 50° Brix cranberry juice concentrate were stored at −20 °C for up to one year. For subsequent experiments, frozen concentrate was melted in a warm water bath immediately prior to diluting to a single-strength juice of 7.5° Brix using ultrapure water.

For the accelerated aging study, four batch replicates of single-strength juice were prepared for each of 13 time points. To accelerate loss of monomeric anthocyanins, juice samples were incubated at 50 ± 4 °C for up to 10 days. At each time point, four samples were removed from the incubator and agitated until the juice appeared homogenous. Samples with 0–7 days accelerated aging were agitated by manually shaking where samples with 8–10 days accelerated aging required vortexing for 15 s. A 93.0 µL sub-sample of each 5 mL sample of whole juice was removed for turbidity analysis. Subsequently, samples of juice were centrifuged to remove any particulate that would interfere with HPLC analysis. Samples with 0–7 days accelerated aging were centrifuged at 3000× *g* for 5.0 min where samples with 8–10 days at 50 °C had to be centrifuged and decanted twice, each time at 3000× *g* for 10.0 min. The supernatant from each sample was immediately analyzed using HPLC, the pH differential method, the percent polymeric color assay, and absorbance spectrophotometry as described below. The precipitate was stored at −20 °C until analysis 14 months later.

Each sample of precipitate was extracted by shaking in 6 mL of methanol on a tube rocker for 24 h. We separated remaining precipitate and methanol extract by centrifuging at 3000× *g* for 10.0 min and stored extract in −20 °C freezer until HPLC analysis. Remaining precipitate was hydrolyzed as described below.

### 2.3. Model System Preparation and Accelerated Aging

Anthocyanins were isolated from cranberry juice concentrate using a published solid phase extraction (SPE) [38,39]. For each purification, a 2000 mg HyperSep C18 SPE cartridge (Thermo Fisher Scientific) was pretreated with 40 mL ethyl acetate, 40 mL methanol, and 40 mL of ultrapure water. Then, cartridges were loaded with 250 µL cranberry juice concentrate diluted in 1.5 mL ultrapure water. Organic acids and sugars were eluted with a wash of 16 mL of 0.01 *v/v*% HCl in ultrapure water, followed by elution of neutral phenols using 80 mL of ethyl acetate. Lastly, cranberry juice anthocyanins were eluted with 16 mL of 0.01 *v/v*% HCl in methanol. Methanol was removed from the purified isolate using a stream of nitrogen and a 25 °C water bath.

For an accelerated aging study paralleling the study of whole juice, four batch replicates of the model system were prepared by dissolving purified cranberry juice anthocyanins in phosphate buffer of pH 2.43, approximating the juice’s pH of 2.44. Phosphate buffer of 2.0 mM was prepared by dissolving KH_2_PO_4_ in ultrapure water and adjusting the pH using dilute HCl. The dried purified anthocyanins from 2 mL of juice concentrate were combined and as much sample was redissolved as possible using 8 rinses of phosphate buffer of 2 mL each with 1 min of vortexing with each rinse as well as shaking on a tube rocker 20 min in a total volume of 18 mL of sample. The model system was isolated from undissolved solids by centrifuging for 5.0 min at 3000× *g*. For each batch replicate of the model system, 9.0 mL of supernatant was placed into glass tubes.

To accelerate loss of anthocyanins in the model system, batch replicates were incubated at 50 ± 4 °C for up to 10 days. At each of 13 time points, the samples were removed from the incubator, vortexed for 15 s, and subsampled before returning to the incubator. A 400 µL subsample was centrifuged at 3000× *g* for 5.0 min to remove any precipitate that would interfere with HPLC analysis. The supernatant of each sample was analyzed using HPLC as described below.

After the final sample collection, precipitate was isolated by centrifuging at 3000× *g* for 5.0 min. The remaining precipitate from each sample was extracted with 3.2 mL methanol by vortexing for 30 s and sonicating at room temperature for 10 min. The remaining precipitate was separated from extract by centrifuging at 3000× *g* for 5.0 min. The methanol extract was analyzed by HPLC and remaining precipitate was stored at −20 °C until hydrolysis using the procedure described below.

### 2.4. Acid Hydrolysis

Precipitate from the whole juice or model system was hydrolyzed using a procedure adapted from Ewald et al. [40]. For each hydrolysis, a hydrolysis solution of 2 mg/mL TBHQ in 2 M HCl in 50/50 methanol/water was freshly prepared. The precipitate from whole juice samples was resuspended in 6 mL of hydrolysis solution and the precipitate from model system samples was resuspended in 2 mL of hydrolysis solution by vortexing for 15 s and sonicating for 10 min. Mixtures were then incubated at 90 °C for 2 h. Prior to HPLC analysis, samples were centrifuged at 3000× *g* for 10.0 min.

### 2.5. Spectrophotometric Analysis Techniques

Samples were analyzed using spectrophotometric techniques previously published with volumes scaled down to analyze samples using a 96-well plate and a SpectraMax 340PC 384 (Molecular Devices, Sunnyvale, CA, USA) [13,41,42]. Total monomeric anthocyanin (MA) content was measured by the pH differential method, AOAC method 2005.02 [41]. Juice supernatant samples were diluted by a factor of 7 using either 25 mM KCl adjusted to pH 1.00 ± 0.05 using HCl or 0.40 M sodium acetate adjusted to pH 4.50 ± 0.05 using HCl so that the 520 nm absorbance (ABS) of samples at pH 1 were between 0.2 and 0.9. The MA content was expressed in micromolar cy3glu equivalents for all samples using Equation (1), where DF is the dilution factor for the sample, ε is the molar extinction coefficient of cy3glu (26,900 L/mol/cm), and b is the path length of 1 cm [42]. PathCheck pathlength measurement technology of the plate reader was used to account for the difference in path length between a 96-well plate and a 1 cm cuvette.

(1) MA=[(ABS520 nm−ABS700 nm)pH 1.0−(ABS520 nm−ABS700 nm)pH 4.5]×DF×(106 μMM)ε×b

The percent polymeric color (%PC) of samples was determined by adapting the bisulfite bleaching method of Wrolstad et al. for a 96-well plate [42]. Briefly, 18.7 µL of freshly prepared 0.2 g/mL potassium metabisulfite or 18.7 µL ultrapure water were mixed with 260 µL diluted juice supernatant. The juice was diluted such that 520 and 420 nm ABS readings were between 0.1 and 0.9, dilution factors of 3 or 5. Polymeric color (Equation (2)) was calculated using absorbance readings of solutions treated with metabisulfite, the color density (Equation (3)) using absorbance readings of solutions analogously diluted with ultrapure water, and the %PC using Equation (4).

(2) $$\mathrm{Polymeric}\;\mathrm{color}=\left[\left({\mathrm{ABS}}_{520\;\mathrm{nm}}-{\mathrm{ABS}}_{700\;\mathrm{nm}}\right)+\left({\mathrm{ABS}}_{420\;\mathrm{nm}}-{\mathrm{ABS}}_{700\;\mathrm{nm}}\right)\right]\times \mathrm{DF}$$

(3) $$\mathrm{Color}\;\mathrm{density}=\left[\left({\mathrm{ABS}}_{520\;\mathrm{nm}}-{\mathrm{ABS}}_{700\;\mathrm{nm}}\right)+\left({\mathrm{ABS}}_{420\;\mathrm{nm}}-{\mathrm{ABS}}_{700\;\mathrm{nm}}\right)\right]\times \mathrm{DF}$$

(4) $$\mathrm{Percent}\;\mathrm{polymeric}\;\mathrm{color}=\frac{\mathrm{Polymeric}\;\mathrm{color}}{\mathrm{Color}\;\mathrm{density}}\times 100$$

Absorbance spectra of 350–750 nm were collected in 1 nm increments using the SpectraMax 340PC 384 plate reader. Batch quadruplicates of whole juice and of juice supernatant were diluted with ultrapure water by 3 to bring the absorbance reading in the operating range of the spectrophotometer. The browning index (BI) was calculated as ABS_520 nm_/ABS_420 nm_ of juice supernatant and turbidity as the absorbance at 650 nm [13].

### 2.6. HPLC Analysis

Each reverse-phase HPLC analysis was performed using a Dionex UltiMate 3000 HPLC equipped with a WPS-3000 analytical autosampler, an LPG-3400 quaternary pump, a DAD-3000 diode array detector, and an FLD 3100 fluorescence detector. Analytes were separated on a Kinetex 5 µm EVO C18, 100 Å, 250 × 4.6 mm column using a binary gradient of formic acid: water (5:95, *v/v*) (A) and methanol (B) published previously [13]. The three-dimensional absorbance data were collected using the diode array detector. Concentrations of gallic acid, PCA, and VA were quantitated using chromatograms at 280 nm, and concentrations of cy3gal, cy3glu, cy3ar, p3gal, p3glu, p3ar, cyanidin, and peonidin were quantitated using chromatograms at 520 nm.

Peaks of the 11 analytes in chromatograms were identified using retention time, spectral characteristics, and standard addition of authentic standards. Analytes were quantitated using external calibration. At least two solvent blanks were measured with each suite of samples at each time point and used to measure the detection limits based on the EPA method detection limit procedure [43]. Detection limits were calculated as 3.3-fold the standard deviation of the signal from the blanks for each analyte and quantitation limits as 10-fold the standard deviation (Supplemental Tables S1 and S2).

### 2.7. Sample Preparation and Analysis by MALDI-TOF MS

Whole juice was freshly prepared and juice supernatant and precipitate with 10 days accelerated aging were prepared as described in the Juice Preparation and Accelerated Aging section. Precipitate from 40 mL of juice was extracted in triplicate, each time by vortexing juice precipitate and 3 mL DMSO for 1 min, sonicating for 5 min, and vortexing for 1 min again, before centrifuging at 3000× *g* for 5.0 min. Prior to purification, one part DMSO extract was mixed with three parts ultrapure water

Prior to Matrix Assisted Laser Desorption/Ionization (MALDI)- Time of Flight (TOF) Mass Spectrometry (MS) analysis, the unaged juice, aged juice supernatant, and aged juice precipitate were fractionated following simplified methods from Krueger et al. [44] We packed glass columns of 1.8 cm diameter with approximately 20 mL of Sephadex LH-20 gel (Santa Cruz Biotechnology, Inc. Dallas, TX, USA) equilibrated for at least 24 h in 30/70 methanol/ultrapure water. Prior to each purification, the column was flushed with at least 50 mL ultrapure water. After samples were loaded, they were eluted with ultrapure water, 50/50 ethanol/methanol, methanol, and then 80/20 acetone/water. Each time, solvent was added until the eluate was visibly clear. For each sample, the 50/50 ethanol/methanol eluate and the methanol eluate were collected as a single fraction (alcohols Sephadex fraction) and the 80/20 acetone/water eluate as a fraction (acetone/water Sephadex fraction). Samples were dried by rotary evaporation.

Samples were analyzed using a Bruker ULTRAFLEX III with SmartBeam laser in positive reflectron mode over the mass range 500–2900 Da. Each sample was mixed with DHB matrix using methanol. PPG 2000 was used for mass calibration via external calibration.

Mass spectra were analyzed using mMass software version 5.5.0. Libraries were constructed on the mMass software with the molecular formulas of monomers of cyanidin- and peonidin-hexosides and -pentosides; homo- and heterodimers of cyanidin- and peonidin-hexosides and -pentosides; pyranoanthocyanins identified in cranberry products [45]; (epi)catechin connected to two units of cyanidin- or peonidin-hexosides or pentosides; proanthocyanidins (PACs); PACs-anthocyanin heteropolymers with one cyanidin- or peonidin-hexoside or -pentoside unit; and PACs-quercetin heteropolymers. More specifically, libraries included PACs of 1–23 (epi)catechin units, PACs with 0–4 A-type linkages, and PACs-anthocyanin polymers where PACs were chemically bonded to anthocyanins both with a CH_3_CH bridge (ethylene cross-linked) and without an ethylene bridge (directly bonded).

### 2.8. Statistical Analyses

Data were reported as the means ± standard deviations. Best-fit linear regressions, two-way ANOVA analyses with Sidak’s multiple comparisons test, unpaired *t* tests, and F tests were performed using GraphPad Prism 9.0.1 (La Jolla, CA, USA). Welch’s correction was used in all *t* tests where an F test showed that data sets had statistically different variances. The Holm–Sidak correction factor was used for multiple *t* tests involving multiple comparisons. For all analyses, statistical significance was defined as *p* < 0.05.

## 3. Results and Discussion

### 3.1. Chemical Composition Cranberry Juice and Model Juice System

The six largest anthocyanin peaks in cranberry juice were quantitated by HPLC-DAD analysis (Table 1 and Supplemental Table S3 and Figures S1–S5 referenced therein). Freshly prepared juice had 46.45 ± 0.04 µM cy3gal (20.87 ± 0.02 mg/L), 4.29 ± 0.01 µM cy3glu (1.928 ± 0.004 mg/L), 81.11 ± 0.05 µM cy3ar (34.01 ± 0.02 mg/L), 46.02 ± 0.04 µM p3gal (21.33 ± 0.02 mg/L), 10.43 ± 0.01 µM p3glu (4.833 ± 0.005 mg/L), and 40.47 ± 0.03 µM p3ar (17.54 ± 0.01 mg/L). These values are congruent with prior literature describing cranberry anthocyanins as mainly cyanidin- and peonidin- galactosides and -arabinosides followed by glucosides [46,47,48,49,50,51,52,53]. Although the model system under-represented total anthocyanin content by approximately 50% because of purification or limited solubility (Table 1), the relative contribution of each of the six anthocyanins to the overall anthocyanin profile was similar (Supplemental Table S4). The model system under-represented p3gal by 4.7% and over-represented cy3ar by 6.7%, and over- or under-represented other anthocyanins to lesser degrees (Supplemental Table S4).

Gallic acid, PCA, and VA have previously been identified in cranberry products and extracts though the concentrations of these compounds in juice are not extensively reported [54,55,56,57]. The freshly prepared juice had 15.9 ± 0.1 µM gallic acid (2.70 ± 0.02 mg/L), 95 ± 2 µM protocatechuic acid (14.6 ± 0.3 mg/L), and 38.6 ± 0.7 µM VA (6.5 ± 0.1 mg/L). As desired, SPE reduced initial gallic acid, PCA, and VA concentrations by between 96 and 99% (Table 1).

Colorimetric analyses showed that this juice was one of high quality for polyphenol content and color quality. The pH differential analysis showed that the cranberry juice had 150 ± 7 µM (or 68 ± 3 mg/L) cyanidin 3-*O*-glucoside equivalents, a high value for cranberry juice at market but not exceptionally high compared to other fruits [13,58]. Treatment with metabisulfite showed that the juice had 20. ± 2%PC, a low value for a juice at market [13]. Finally, the juice had a BI of 2.1 ± 0.2, an exceptionally high value for a juice at market [13].

### 3.2. Anthocyanin Loss Rates

When subjected to storage at 50 °C, soluble anthocyanins in whole juice and the model system followed a pseudo-first-order loss rate as others have observed (Table 1, Supplemental Figures S1 and S3) [59]. Overall, the anthocyanins degraded more slowly in the model system than in whole juice. Both systems followed a pseudo-first-order model, so the difference in rate was likely partially explained by the initial concentrations of anthocyanins being lower in the model system than in the whole juice. However, even at similar concentrations of anthocyanin, the instantaneous rate of change of the anthocyanin in the model system was less than that in the whole juice (data not shown), so concentration alone does not account for differences in loss rates of anthocyanins. Interactions with other components in the whole juice likely contributed to loss of anthocyanins. This supposition agreed with our observation of increasing amounts of pigmented precipitate from whole juice during accelerated aging.

Additionally, in the whole juice, the magnitude of rate constants followed the order p3ar > cy3ar > p3gal > p3glu > cy3gal > cy3glu, where in the model system, the magnitude of the rate constants followed the order p3ar > cy3ar > p3gal > cy3gal > p3glu. Given that initial concentration could change relative loss rate, the inversion of p3glu and cy3gal could be because the model system over-represented cy3gal concentration and under-represented p3glu concentration relative to other anthocyanins (Supplemental Table S4). The fact that all anthocyanins had slower loss rates in model system than in juice supernatant and relative loss rates of individual anthocyanins differed slightly confirmed our hypothesis that loss processes differ between whole juice and related model systems, though only slightly.

### 3.3. Loss Rate Based on Anthocyanidin

Each peonidin-glycoside had a larger magnitude of rate constant and shorter half-life than its corresponding cyanidin-glycoside in both the whole juice and model system (Table 1). Generally, anthocyanidins that are alkoxylated rather than hydroxylated (i.e., peonidin vs. cyanidin) are more stable to various degradation mechanisms, but not across all conditions [18,60,61]. Our data were consistent with Trost et al. who reported higher degradation rate constants for peonidin-glycosides than for corresponding cyanidin-glycosides in blueberry-aronia nectar stored at 30 °C and with Attoe and von Elbe who reported higher degradation rate constants for peonidin-glycosides than for corresponding cyanidin-glycosides purified from cranberry, dissolved in phosphate buffer of pH 2.5, and stored at 40 °C [62,63]. Notably, Attoe and von Elbe reported higher degradation rates for cyanidin-glycosides than for corresponding peonidin-glycosides when incubated at 55 °C. Comparing the present study with Attoe and von Elbe’s implies that there is a change in the processes by which soluble free anthocyanins are lost between 50 and 55 °C.

### 3.4. Loss Rate Based on Glycoside

In both the model system and the whole juice, anthocyanidin-arabinosides had rate constants of higher magnitude than anthocyanidin-galactosides. Further, in the whole juice, the anthocyanidin-galactosides had rate constants of higher magnitude than anthocyanidin-glucosides, and in the model system, peonidin-galactoside had a rate constant of higher magnitude than peonidin-glucoside. These observations are consistent with literature describing anthocyanidin-pentosides as degrading more readily than -hexosides [16,62,63,64,65], and anthocyanidin-galactosides degrading more quickly than -glucosides, likely because of added steric hinderance [62,64,65,66].

Interestingly, the relative importance of the constituent anthocyanidin as compared to the constituent glycoside to the loss rate of each anthocyanin differed between the model system and whole juice. In the model system, rate constants decreased in magnitude in the order anthocyanidin-arabinoside > anthocyanidin-galactoside > anthocyanidin-glucoside, with the rate constant for cyanidin-glucoside below the limit of quantitation. Others have similarly found anthocyanins isolated from blueberry deglycosylated in the order arabinosides > galactosides > glucosides regardless of constituent anthocyanidin [65], and cranberry anthocyanins in phosphate buffer degraded in the order arabinoside > galactoside regardless of anthocyanidin at both 40 °C and 55 °C in both light and dark conditions [63].

In contrast, in the whole cranberry juice, rate constants decreased in magnitude in the order anthocyanidin-arabinoside > peonidin-hexoside > cyanidin-hexoside. Unlike in the model system, whether the anthocyanin was a galactoside or a glucoside had less effect on the loss rate than whether the anthocyanin was a cyanidin or peonidin. Trost et al. observed a similar phenomenon when measuring anthocyanins in blueberry-aronia nectar: that arabinosides generally had higher *k* values than galactosides and glucosides where within galactosides or glucosides the *k* values were organized not by sugar but by number of oxygenated substituents on the B ring of the anthocyanidin [62]. In their data, malvidin 3-*O*-galactoside even had a higher *k* than cyanidin 3-*O*-arabinoside.

The results of the present study extend these previous observations with a direct comparison of a whole juice and a system of isolated anthocyanins in buffer. Here, the difference in relative importance of anthocyanidin as compared to glycoside likely is not particular to the fruit from which the anthocyanins are isolated but instead depends on the matrix. Future studies should corroborate this finding and expand it to include the analysis of more constituent anthocyanidins in model systems, to include additional pentosides in the analysis of model systems and whole juices, and to determine which component(s) of juice affect the relative stability of anthocyanins.

### 3.5. Implications of Anthocyanin Loss on Bioactivity of Juice

It is difficult to attribute the specific impact of anthocyanin loss on the putative health effects of cranberry juice consumption because of the presence of phenolic acids, other flavonoids, and proanthocyanidins in the juice matrix. Prior research implies that anthocyanins have greater antioxidant efficacy than their corresponding phenolic acids [10,11]. Furthermore, anthocyanins exhibit specific anti-inflammatory effects as reviewed by others [67]. Therefore, it is reasonable to assume that chemical degradation or redistribution of anthocyanins and other polyphenols in juice could lead to a reduction in the putative health benefits associated with the regular consumption of cranberry juice.

### 3.6. Formation of Hydroxybenzoic Acids

PCA is a degradation product of cyanidin-glycosides (cy3gly) and VA a degradation product of peonidin-glycosides (p3gly). The concentrations of PCA and VA in the model system and the whole juice increased linearly, or followed a pseudo-zeroth-order reaction model, similar to other studies [17,18,19,68]. In both systems, PCA had a higher rate constant and formed more quickly than VA. This was probably because there was a higher concentration of cyanidin-glycosides than peonidin-glycosides in both systems.

**Figure 1:**
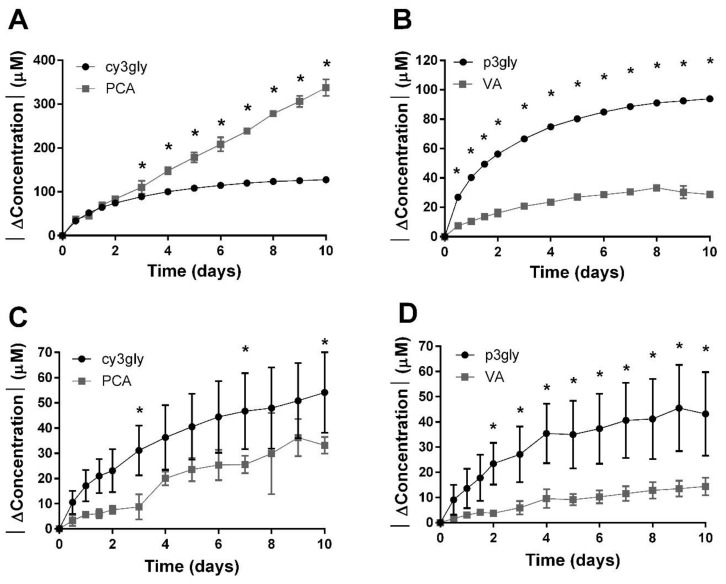
Accelerated aging of cranberry juice or isolated anthocyanins at 50 °C alters soluble anthocyanins and hydroxybenzoic acids. Data are the absolute value of concentration change from Time 0, as the means ± standard deviations. (**A**) Cyanidin 3-*O*-glycosides (cy3gly) and protocatechuic acid (PCA) in whole juice. (**B**) Peonidin 3-*O*-glycosides (p3gly) and vanillic acid (VA) in whole juice. (**C**) Cy3gly and PCA in anthocyanin isolate. (**D**) P3gly and VA in anthocyanin isolate. Cy3gly is the sum of cyanidin 3-*O*-galactoside, cyanidin 3-*O*-glucoside, and cyanidin 3-*O*-arabinoside, while p3gly is the sum of peonidin 3-*O*-galactoside, peonidin 3-*O*-glucoside, peonidin 3-*O*-arabinoside. For those time points indicated (*), the change in total anthocyanin concentration and change in corresponding hydroxybenzoic acid concentration are statistically different according to two-way ANOVA with Sidak’s multiple comparisons test, where *p* < 0.05. For (**A**,**B**), *p* < 0.0001 for Time, ΔConcentration, and their interaction; for (**C**), *p* < 0.001 for Time, *p* = 0.0318 for ΔConcentration, and *p* = 0.0038 for their interaction; and for (**D**), *p* < 0.0001 for Time, *p* = 0.0097 for ΔConcentration, and *p* < 0.0001 for their interaction.

To measure the utility and validity of the concentration of PCA as a tracer for loss of cyanidins, we compared the increase in PCA concentration between initial value and concentration at each time point to the decrease in the sum of cy3gal, cy3glu, and cy3ar concentrations between initial values and sum of concentrations at each time point. Similarly, we compared the increase in VA concentration from initiation of aging to the decrease in the sum of p3gal, p3glu, and p3ar concentrations from initial values (Figure 1). Generally, the increase in the concentration of hydroxybenzoic acids under-represented the decrease in the concentration of anthocyanins, indicating that other loss mechanisms are important. More specifically, in the model system, PCA formation accounted for between 26% and 82% of the cy3gly lost and VA for between 19% and 30% of the p3gly lost. In the whole juice, PCA formation accounted for between 87% and 265% of cy3gly lost and VA for between 26% and 37% of p3gly lost.

Strikingly, the change in the PCA concentration of whole juice over-represented the change in cy3gly concentration within 2 days of accelerated aging. This was likely because cranberries have an appreciable concentration of quercetin glycosides which have the same B ring structure as cyanidins and could similarly form PCA via scission [69,70,71,72,73,74,75,76]. Notably, PCA did not similarly over-represent cy3gly in the model system where purification would be expected to remove quercetin glycosides, nor did VA similarly over-represent p3gly as isorhamnetin is not abundant in cranberry. For the hydroxybenzoic acid that forms from the B ring of anthocyanins to be an effective tracer of anthocyanin loss, the relative rates of degradation of anthocyanidins and their corresponding flavonols require further characterization.

Interestingly, for the first 2 days of accelerated aging, the change in the concentration of PCA was a good approximation for the change in the concentration of cy3gly in whole juice. In fact, the two values are not statistically different until 3 days of accelerated aging. This implies that there is a lag in the degradation of quercetins in whole juice compared to cyanidins in whole juice or that the degree of degradation of quercetins to PCA initially approximated the degree of degradation of cyanidins via other mechanisms. The former explanation is in keeping with a recent study by Kellil et al., who found that thermal degradation of quercetin best follows a sigmoidal curve [75]. In their analysis, the length of time of the initially slow loss rate of quercetin depended on the concentration of the various antioxidants they added and the pH of the system. Continued analysis would be useful to help understand the interconnected loss processes of anthocyanins and structurally related flavonols, especially when and how each is best represented by the hydroxybenzoic acid formed from their B rings.

### 3.7. Quantitative Summary of Loss of Cyanidin-Glycosides and Peonidin-Glycosides

Table 2 summarizes the loss processes of anthocyanins in cranberry juice and the model system. Because the products of C-ring scission of cyanidins, quercetins, and (epi)catechins all include PCA, and because cyanidin-glycosides and (epi)catechins all form cyanidin upon hydrolysis, the increase in soluble PCA concentration and the increase in hydrolysable cyanidin concentration over-represented cyanidin-glycosides lost to whole cranberry juice. The importance of quercetins to total change in PCA concentration in cranberry juice supernatant was corroborated by the fact that the percent of change in cyanidin-glycoside concentration attributable to the increase in PCA concentration was statistically higher in whole juice than in model system (Table 2 and Supplemental Figures S4–S7 and Tables S1 and S2 referenced therein). Similarly, the statistically significant difference between percent cyanidin-glycosides attributable to hydrolysable cyanidin in juice precipitate and model system precipitate corroborated the importance of (epi)catechins to measurement of hydrolysable cyanidin in cranberry juice. Based on the model system, soluble PCA formation accounted for 70 ± 20% of cyanidin-glycosides lost, and formation of insoluble materials accounted for 16 ± 6% of cyanidin-glycosides lost.

Unlike cyanidin-glycoside, peonidin-glycoside loss processes in whole cranberry juice were not confounded by other polyphenols. After 10 days at 50 °C, the increase in soluble VA concentration accounted for 31 ± 2% of soluble peonidin-glycosides lost in whole juice and 35 ± 5% in model system. Formation of insoluble materials accounted for 3 ± 1% of soluble peonidin-glycosides lost in whole juice and 1.6 ± 0.8% in model system. Importantly, the similarity in values of peonidin-glycoside loss processes between model system and whole juice supports the model system as a useful tool for closely approximating anthocyanin loss processes and identifying important interfering chemical species, like those which affected quantitation of cyanidin-glycoside loss processes.

These data confirmed our initial hypothesis that the insoluble phase accumulates anthocyanins. Prior studies have neglected the insoluble phase as a route of anthocyanin loss, given the difficulty in characterizing its polyphenol content [13,18,19]. The magnitude of loss of anthocyanins is small: 3 ± 1% if peonidin-glycosides lost to precipitate in whole juice is used as representative of total loss, or between 1.6 ± 0.8% and 16 ± 6% if cyanidin-glycosides and peonidin-glycosides lost to precipitate in model system is used as a representative of total loss. Loss processes could vary between juices prepared by different manufacturing process. Nonetheless, the presence of anthocyanins in the precipitate of even highly clarified juice like the one used in this study is potentially pertinent for industrial scale production of juices and the byproducts in juice production.

### 3.8. Precipitate Analysis

**Figure 2:**
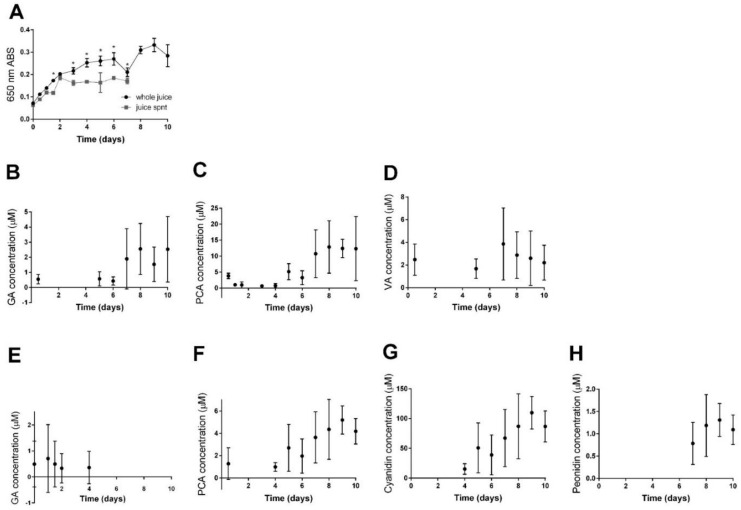
Time course of precipitate formation in cranberry juice and precipitate composition during accelerated aging at 50 °C. (**A**) Light scattering (650 nm) in whole juice and centrifuged juice supernatant (spnt). Absorbances of whole juice and juice spnt were statistically different at indicated (*) time points between 0 and 7 days of accelerated aging according to two-way ANOVA with Sidak’s multiple comparisons test (*p* < 0.0001 for Time, *p* < 0.0001 for Sample type, and *p* < 0.0001 for their Interaction). (**B**–**D**) Polyphenols detected in methanol extract of precipitate. (**B**) Gallic acid. (**C**) Protocatechuic acid. (**D**) Vanillic acid. (**E**–**H**) Polyphenols detected in hydrolysate of precipitate. (**E**) Gallic acid. (**F**) Protocatechuic acid. (**G**) Cyanidin. (**H**) Peonidin. Data points or analytes not shown in **B**–**H** were below limits of quantitation. Detection and quantitation limits are included in Supplemental Table S1.

Clarified cranberry juice held at 50 °C progressively formed pigmented precipitate (Figure 2A and Supplemental Figure S10). Some of this precipitate, especially after 3 days at 50 °C, was isolated via centrifugation (Figure 2A). This isolable fraction represents sedimentable cloud or haze [29]. Additional centrifugation was necessary to remove visible haze after 8 days of accelerated aging. Notably, though haze was not perceptible as particles that would interfere with HPLC analysis, light scattering material measurable as 650 nm ABS remained in cranberry juice after centrifugation.

HPLC was used to characterize quantitatively the anthocyanins and components with anthocyanidin substituents within whole cranberry juice precipitate. Methanol extracts of aged juice precipitate had detectable though not quantifiable levels of free anthocyanins (example chromatograms in Supplemental Figure S6B, LODs and LOQs in Supplemental Table S1). Because monomeric anthocyanins tend to be highly soluble in methanol, the fact that the methanol extract of juice precipitate had exceedingly low levels of anthocyanins implies either that there were very few free anthocyanins present or that anthocyanins were bound to materials in precipitate that were insoluble in methanol. Hydrolysis showed that juice precipitate had increasing amounts of hydrolysable cyanidin and peonidin. Amount of hydrolysable cyanidin varied between 4.1 µM and 110 ± 30 µM cyanidins in whole juice, though this was likely an over-representation because of the hydrolysis of proanthocyanidins (Supplemental Table S1, Figure 2G); amount of hydrolysable peonidin varied between 0.17 and 1.3 ± 0.4 µM peonidins (Supplemental Table S1, Figure 2H).

Additionally, MALDI-TOF was used to characterize qualitatively and semi-quantitatively the polyphenol components, especially anthocyanin-PAC heteropolymers, of freshly prepared cranberry juice and supernatant and precipitate of juice after 10 days at 50 °C (Supplemental Figure S11). MALDI-TOF confirmed the presence of anthocyanins in the form of anthocyanin-PAC polymeric colors in all fractions (Table 3 and Supplemental Table S5). Interestingly, anthocyanin-PACs with no ethylene bridge made up more of the anthocyanin-PACs in aged juice precipitate than in aged juice supernatant (Supplemental Table S6). This is apparent in the fact that only 20% of the intensity of tentatively identified anthocyanin-PACs peaks was from anthocyanin-PACs without an ethylene bridge for the alcohols Sephadex fraction as compared to all the other Sephadex fractions from aged juice including those of precipitate where anthocyanin-PACs without an ethylene bridge made up 42–43% of the intensity of tentatively identified anthocyanin-PACs peaks.

### 3.9. Polymeric Color Analyses

The %PC assay and MALDI-TOF analysis confirmed the presence of polymeric pigments and provided qualitative and semi-quantitative characterization of those pigments. Polymeric colors made up 20 ± 2% of the juice’s overall pigmentation and that value doubled in approximately 2.8 days’ time (Table 1). This is an especially short half-life compared to the 9- to 133-day half-lives of juices similarly incubated at 50 °C previously reported [13]. As summarized in Table 3 and fully enumerated in Supplemental Table S5, MALDI-TOF analysis confirmed the presence of polymeric pigments in the form of anthocyanins covalently bound to proanthocyanidins in freshly prepared juice and in the supernatant and precipitate of juice after 10 days accelerated aging [39]. We did not see evidence of anthocyanin-anthocyanin dimers, anthocyanin-anthocyanin-PACs polymers, nor the pyranoanthocyanins previously identified in cranberry extracts [45]. These compounds along with others such as xanthylium ions are important to consider as they interfere with measurement of anthocyanin-PACs by the %PC assay. It is possible that MALDI-TOF analysis did not detect the presence of any such analytes because they eluted with the aqueous fraction during Sephadex LH-20 or because they did not desorb and ionize well, the latter being an especially important consideration for multiply-charged polymers with multiple anthocyanin groups.

Cranberry anthocyanins were covalently bonded both with and without ethylene bridges to PACs of between one and four (epi)catechin units with zero to two A-type bonds between (epi)catechin units (Table 3). Although A-type bonds were observed, we did not account for the natural abundances of isotopes of carbon, hydrogen, and oxygen to measure the percentage of A- and B-type bonds as others have done [77,78,79]. Additionally, a narrower range of degrees of polymerization of PACs bound to anthocyanins and narrower range of number of A-type linkages were detected than Krueger et al. previously observed [44]. Plausibly, this is because they analyzed whole cranberry fruit extract rather than cranberry juice implying that the manufacture of cranberry juice can decrease the complexity of cranberries’ polymeric color profile.

On the basis of MALDI-TOF signal, the polymeric pigments in cranberry juice were mainly peonidins (Supplemental Table S6). More specifically, peonidin-glycoside-PAC heteropolymers made up between 72% and 96% of isolable polymeric colors based on the identified anthocyanin-PAC peak intensities in the alcohols Sephadex fraction of freshly prepared whole juice and the acetone/water fraction of aged juice precipitate. This is particularly striking because identified peonidin-glycosides contributed less to total soluble anthocyanins than identified cyanidin-glycosides. Specifically, peonidin-glycosides made up between 40.8 ± 0.2% and 42.36 ± 0.02% of soluble anthocyanins in cranberry juice with up to 10 days at 50 °C. We also observed higher proportions of peonidin in aged juice precipitate than in aged juice supernatant and in acetone/water Sephadex fractions than in alcohols Sephadex fraction, but these differences were less pronounced and compelling (Supplemental Table S6).

## 4. Conclusions

Storage of cranberry juice at 50 °C depletes soluble monomeric anthocyanins, an important class of health-promoting antioxidant compounds. In this study, the fate of soluble, precipitated, and hydrolysable phenolics in aged cranberry juice supports the hypothesis that anthocyanin loss processes in juice are not well accounted for and include not only the formation of hydroxybenzoic acids but also loss to precipitation. The single largest sink was formation of hydroxybenzoic acids, but PCA and VA accounted for a minority of the anthocyanin loss. Precipitate formation did explain between 3 ± 1% and 16 ± 6% loss of anthocyanins, mostly as polymeric colors. Furthermore, anthocyanin loss processes differed between whole juice and a simplified system of cranberry juice anthocyanins in buffer. Soluble PCA formation and hydrolysable cyanidin were different between whole juice and the model system. The differences indicated the utility of model systems for identifying interfering chemical species rather than the inaccuracy of model systems to whole juice. Isolated cranberry juice anthocyanins in buffer under-represented the rate constants of individual anthocyanins’ loss or hydroxybenzoic acids’ formation. Additionally, the relative importance of constituent anthocyanidin or glycoside to an anthocyanin’s loss rate differed between model system and whole juice. Finally, non-specific spectrophotometric methods typically used for juice quality analysis do not accurately and completely capture anthocyanin loss processes. This was true because the pH differential method under-estimated the concentration of anthocyanins present in freshly prepared cranberry juice from frozen concentrate, and thus under-estimated the loss rate of anthocyanins in juice. Analysis of %PC supplied useful though limited information. It semi-quantitatively corroborated the detailed qualitative analysis of anthocyanin-PAC formation supplied by MALDI-TOF, but because the amount of polymeric pigment in each sample was normalized to the total amount of pigment, it could not quantitate the change in anthocyanin-PACs in cranberry juice. BI can be used to estimate juice pigment quality, but does not quantitatively correlate with loss of anthocyanins nor formation of polymeric pigments.

## Figures and Tables

**Table 1:** Polyphenol content of cranberry juice supernatant or isolated cranberry juice anthocyanins in buffer over 10 days accelerated aging at 50 °C ^a^.

Analyte	Initial Value ^b^	Pseudo-Reaction Order ^c^	*k* ^d^	t_1/2_ (Days) ^e^
cranberry juice supernatant				
gallic acid	15.9 ± 0.1	0th	6.0 ± 0.1	1.32 ± 0.03
protocatechuic acid	95 ± 2	0th	32.5 ± 0.5	1.46 ± 0.04
vanillic acid	38.6 ± 0.7	0th	2.8 ± 0.2	6.9 ± 0.5
cyanidin 3-*O*-galactoside	46.45 ± 0.04	1st	−0.290 ± 0.002	−2.39 ± 0.02
cyanidin 3-*O*-glucoside	4.29 ± 0.01	1st	−0.281 ± 0.002	−2.47 ± 0.02
cyanidin 3-*O*-arabinoside	81.11 ± 0.05	1st	−0.359 ± 0.003	−1.93 ± 0.02
peonidin 3-*O*-galactoside	46.02 ± 0.04	1st	−0.311 ± 0.003	−2.23 ± 0.02
peonidin 3-*O*-glucoside	10.43 ± 0.01	1st	−0.296 ± 0.0003	−2.34 ± 0.02
peonidin 3-*O*-arabinoside	40.47 ± 0.03	1st	−0.373 ± 0.003	−1.86 ± 0.02
anthocyanins (sum)	228.7 ± 0.1	1st	−0.327 ± 0.003	−2.12 ± 0.02
anthocyanins (pH differential)	150 ± 7	1st	−0.230 ± 0.004	−3.00 ± 0.05
polymeric color (%)	20. ± 2%	0th	3.5 ± 0.2	2.8 ± 0.1
browning index	2.1 ± 0.2	2nd	0.085 ± 0.002	5.7 ± 0.7
isolated cranberry juice anthocyanins				
gallic acid	0.7 ± 0.3	0th	0.22 ± 0.02	1.5 ± 0.7
protocatechuic acid	2.0 ± 0.4	0th	3.6 ± 0.3	0.27 ± 0.06
vanillic acid	0.5 ± 0.2	0th	1.4 ± 0.1	0.16 ± 0.06
cyanidin 3-*O*-galactoside	28 ± 7	1st	−0.139 ± 0.007	−5.0 ± 0.3
cyanidin 3-*O*-arabinoside	34 ± 8	1st	−0.197 ± 0.009	−3.5 ± 0.2
peonidin 3-*O*-galactoside	28 ± 6	1st	−0.15 ± 0.02	−4.6 ± 0.7
peonidin 3-*O*-glucoside	8 ± 2	1st	−0.12 ± 0.02	−6 ± 1
peonidin 3-*O*-arabinoside	21 ± 7	1st	−0.20 ± 0.02	−3.4 ± 0.4
anthocyanins (sum)	120 ± 30	1st	−0.159 ± 0.008	−4.4 ± 0.2

^a^ Plots of data and linear regressions are shown in Supplemental Figures S1–S3. Example chromatograms are shown in Supplemental Figures S4 and S5. ^b^ These are the initial concentrations of chemical species or values measured in the sample prior to accelerated aging. The average values are reported with standard deviations of batch triplicate or quadruplicate samples. Concentrations of individual chemicals or sums of those chemicals were measured and are reported in micromolar units. Total monomeric anthocyanin as measured by the pH differential method was calculated in micromolar cyanidin 3-*O*-glucoside equivalents. Percent polymeric color is a dimensionless quantity though reported as a percentage as is custom. The browning index is a dimensionless quantity. ^c^ We calculated the linear least square regression of the concentrations of chemical species or reported PC measurement for pseudo-zeroth-order analyses, the linear least square regression of the natural logarithm of the concentrations of chemical species for pseudo-first-order analyses, and the linear least square regression of ABS_420 nm_/ABS_520 nm_ (the inverse of BI) for the pseudo-second-order analysis of BI. ^d^ *k*, rate constants. These rate constants are the slopes of the calculated linear regressions. Values are reported with standard deviations from the regression analysis. For pseudo-zeroth-order analyses, the units for the rate constant are (measured value/time), so (µM/day) for individual chemical species or (day^−1^) for polymeric color. For pseudo-first-order analyses, the rate constants are in (day^−1^). For the pseudo-second-order analysis of the browning index, the browning index is a dimensionless quantity, so the rate constant is in (day^−1^). ^e^ t_1/2_, half-life. We calculated and reported half-lives as (initial value/2/*k*) for pseudo-zeroth-order analyses, (ln [2]/*k*) for pseudo-first-order analyses, and (k*initial value)^−1^ for the pseudo-second-order model. Values are reported with standard deviations propagated from the standard deviations of rate constants and measured initial value.

**Table 2:** Concentrations of anthocyanins, products of their loss processes, and normalized changes in the concentration of anthocyanin products as percentages with accelerated aging at 50 °C in cranberry juice supernatant and in isolated cranberry juice anthocyanins in buffer ^a^.

Juice Fraction	Component	Concentration in Fresh Sample (µM) ^b^	Concentration after 10 Days at 50 °C (µM) ^b^	ΔConcentration (µM)	$\frac{\Delta Concentration}{\Delta Concentration\;Anthocyanidin}$
whole cranberry juice					
soluble components	cyanidin glycosides	131.84 ± 0.08	4.3 ± 0.4 ^c^	−127.5 ± 0.4	100%
	protocatechuic acid	95 ± 2	430 ± 20 ^c^	340 ± 20	260 ± 10% ^e^
extract from precipitate	cyanidin glycosides	<12	<12	0	0%
	protocatechuic acid	0.5 ± 0.6	10 ± 10 ^c^	12 ± 9	9 ± 7%
hydrolysate of precipitate	cyanidin	<12	90 ± 30 ^c^	90 ± 30	70 ± 20% ^e^
	protocatechuic acid	<0.46	4 ± 1 ^c^	4 ± 1	3.2 ± 0.9%
soluble components	peonidin glycosides	96.91 ± 0.07	3.0 ± 0.3 ^d^	−93.9 ± 0.3	100%
	vanillic acid	38.6 ± 0.7	67 ± 2 ^d^	29 ± 2	31 ± 2%
extract from precipitate	peonidin glycosides	<0.50	<0.50	0	0%
	vanillic acid	<0.27	2 ± 1 ^d^	2 ± 1	2 ± 1%
hydrolysate of precipitate	peonidin	<0.51	1.1 ± 0.3 ^c^	1.0 ± 0.3	1.1 ± 0.4%
	vanillic acid	<0.27	<0.27	0	0%
isolated cranberry juice anthocyanins					
soluble components	cyanidin glycosides	70 ± 20	11 ± 2 ^c^	−50 ± 20	100%
	protocatechuic acid	2.0 ± 0.4	35 ± 3 ^c^	33 ± 3	70 ± 20% ^e^
extract from precipitate	cyanidin glycosides	<11	<11	0	0%
	protocatechuic acid	<0.39	0.6 ± 0.5	0.6 ± 0.5	1.0 ± 0.5%
hydrolysate of precipitate	cyanidin	<1.3	6 ± 5	6 ± 5	9 ± 6% ^e^
	protocatechuic acid	<2.6	3.6 ± 0.5 ^c^	3.1 ± 0.5	6 ± 1%
soluble components	peonidin glycosides	50 ± 20	11.8 ± 0.8 ^c^	−40 ± 20	100%
	vanillic acid	0.5 ± 0.2	15 ± 3 ^c^	14 ± 3	35 ± 5%
extract from precipitate	peonidin glycosides	<12	<12	0	0%
	vanillic acid	<0.33	<0.33	0	0%
hydrolysate of precipitate	peonidin	<0.70	0.8 ± 0.6	0.7 ± 0.6	1.6 ± 0.8%
	vanillic acid	<0.68	<0.68	0	0%

Data are the mean ± standard deviation of *n* = 4 experiments. ^a^ Example chromatograms are shown in Supplemental Figures S4–S7. ^b^ Values below the quantitation limits (LOQ) are reported as < LOQ. Detection and quantification limits are included in Supplemental Tables S1 and S2. ^c^ Concentrations in samples with no accelerated aging or after 10 days at 50 °C are statistically different based on an unpaired *t* test with Welch’s correction (*p* < 0.05). ^d^ Concentrations in fresh juice and in samples after 10 days accelerated aging at 50 °C are statistically different based on an unpaired *t* test (*p* < 0.05). For these, F test showed the variances were not statistically different (*p* < 0.05). ^e^ The percentage of corresponding group of anthocyanins lost to the identified analyte is statistically different between whole cranberry juice and isolated cranberry juice anthocyanins based on a two-way ANOVA with Sidak’s multiple comparisons test (*p* < 0.0001 for row factor, column factor, interaction of row and column factor, and adjusted *p* < 0.0001 for comparison of row means for soluble protocatechuic acid and hydrolysable cyanidin).

**Table 3:** Anthocyanin-proanthocyanidins tentatively identified in MALDI-TOF mass spectra of cranberry juice with 0 or 10 days accelerated aging at 50 °C ^a^.

Juice Fraction	Sephadex Fraction ^b^	Anthocyanin	# (Epi)catechin Units	# A-Type Linkages	Anthocyanin-Procyanidin Connection
unaged juice					
whole juice	alcohols	cyanidin-hexoside	1–4	0–2	ethylene cross-linked, directly bonded
		cyanidin-pentoside	1–3	0–1	ethylene cross-linked
		peonidin-hexoside	1–4	0–2	ethylene cross-linked, directly bonded
		peonidin-pentoside	1 and 3	0–1	ethylene cross-linked
	acetone/water	cyanidin-hexoside	2	0	ethylene cross-linked
		cyanidin-pentoside	1	0	ethylene cross-linked
		peonidin-hexoside	1–2	0–1	directly bonded
		peonidin-pentoside	2	0–1	ethylene cross-linked
aged juice					
supernatant ^c^	alcohols	cyanidin-hexoside	2–3	1–2	ethylene cross-linked
		cyanidin-pentoside	2	1	ethylene cross-linked
		peonidin-hexoside	2–3	1–2	ethylene cross-linked, directly bonded
		peonidin-pentoside	2	0–1	ethylene cross-linked
	acetone/water	cyanidin-hexoside	2	0	ethylene cross-linked
		cyanidin-pentoside	1	0	ethylene cross-linked
		peonidin-hexoside	2	0–1	ethylene cross-linked, directly bonded
		peonidin-pentoside	2	0–1	ethylene cross-linked,
precipitate ^c^	alcohols	cyanidin-hexoside	2	0	ethylene cross-linked
		peonidin-hexoside	2	1	directly bonded
		peonidin-pentoside	2	0–1	ethylene cross-linked
	acetone/water	cyanidin-hexoside	2	1	ethylene cross-linked
		cyanidin-pentoside	1	0	ethylene cross-linked
		peonidin-hexoside	2	1	directly bonded
		peonidin-pentoside	2	0–1	ethylene cross-linked

^a^ Masses and molecular formulas of tentatively identified peak are in Supplemental Table S5. ^b^ Samples for MALDI-TOF analysis were from 50/50 ethanol/methanol and methanol elution or 80/20 acetone/water elution from Sephadex LH-20 as explained in the Materials and Methods. ^c^ Supernatant and precipitate of juice were separated via centrifugation and precipitate was extracted with dimethyl sulfoxide

## Data Availability

The data presented in this study are available in article and supplementary material.

## Supplementary Materials

The following are available online at https://www.mdpi.com/article/10.3390/antiox10111788/s1, Table S1. Quantitated lower limits of detection (LOD) and quantitation (LOQ) of analytes in cranberry juice supernatant and precipitate extract and hydrolysate; Table S2: Quantitated lower limits of detection (LOD) and quantitation (LOQ) of analytes in isolated cranberry juice anthocyanins, extract of precipitate of isolated anthocyanins, and hydrolysate of precipitate of isolated anthocyanins; Figure S1: The concentration of (A) gallic acid, (B) protocatechuic acid, (C) vanillic acid; natural logarithm of the concentration of (D) cyanidin 3-*O*-galactoside, (E) cyanidin 3-*O*-glucoside, (F) cyanidin 3-*O*-arabinoside, (G) peonidin 3-*O*-galactoside, (H) peonidin 3-*O*-glucoside, (I) peonidin 3-*O*-arabinoside; and (J) the natural logarithm of the sum of the concentration of the six aforementioned anthocyanins in cranberry juice supernatant as measured by HPLC during accelerated aging at 50 °C; Figure S2: (A) Natural logarithm of total anthocyanin content (MA) as measured by the pH differential method, (B) percent polymeric color (%PC), and (C) the inverse of the browning index (BI) of cranberry juice supernatant during accelerated aging at 50 °C; Figure S3: Concentration of (A) gallic acid, (B) protocatechuic acid, (C) vanillic acid; natural logarithm of the concentration of (D) cyanidin 3-*O*-galactoside, (E) cyanidin 3-*O*-arabinoside, (F) peonidin 3-*O*-galactoside, (G) peonidin 3-*O*-glucoside, (H) peonidin 3-*O*-arabinoside; and (I) the natural logarithm of the sum of concentration cyanidin and peonidin 3-*O*-galactoside, -glucoside, and -arabinoside of isolated cranberry juice anthocyanins as measured by HPLC during accelerated aging at 50 °C; Figure S4: Example RP-HPLC chromatograms of freshly prepared juice supernatant (t0) and juice supernatant after 10 days at 50 °C (d10); Figure S5: Example RP-HPLC chromatograms of isolated cranberry juice anthocyanins (t0) and isolated cranberry juice anthocyanins after 10 days at 50 °C (d10); Table S3: Peak numbers in example chromatograms and retention time of analytes in HPLC analyses; Table S4: Percent of each anthocyanin or class of anthocyanins relative to total measured anthocyanin concentration in cranberry juice supernatant and in soluble isolated cranberry juice anthocyanins. Figure S6: Example RP-HPLC chromatograms of methanol extract of precipitate from freshly prepared cranberry juice (t0) and methanol extract of precipitate from cranberry juice after 10 days at 50 °C (d10); Figure S7: Example RP-HPLC chromatograms of hydrolysate of precipitate from freshly prepared cranberry juice (t0) and hydrolysate of precipitate from cranberry juice after 10 days at 50 °C (d10); Figure S8: Example RP-HPLC chromatograms of methanol extract of precipitate from isolated cranberry juice anthocyanins after 10 days at 50 °C; Figure S9: Example RP-HPLC chromatograms of hydrolysate of precipitate from isolated cranberry juice anthocyanins after 10 days at 50 °C; Figure S10: Photograph of pigmented samples analyzed via MALDI-TOF; Figure S11: MALDI-TOF mass spectra of freshly prepared juice samples and samples of juice after 10 days accelerated aging at 50 °C all purified by Sephadex LH-20 chromatography; Table S5: Analytical parameters of MALDI-TOF peaks identified as anthocyanin-procyanidins of cranberry juice, freshly prepared or after 10 days accelerated aging at 50 °C; Table S6: Intensities by constituent anthocyanidin, constituent glycoside, or constituent anthocyanin-proanthocyanidin linkage as percent of total intensity of tentatively identified anthocyanin-proanthocyanidin MALDI-TOF peaks within each analyzed Sephadex fraction.
