# Supplementary material for: Cranberry (Vaccinium macrocarpon) Juice Precipitate Pigmentation Is Mainly Polymeric Colors and Has Limited Impact on Soluble Anthocyanin Loss
Source: Antioxidants (Basel). 2021 Nov 9;10(11):1788. doi: 10.3390/antiox10111788 (PMC8614759; doi:10.3390/antiox10111788)
Supplement: Supplementary file 1 [file antioxidants-10-01788-s001.zip › antioxidants-1426635-supplementary.pdf]

Supplemental Table S1. Quantitated lower limits of detection (LOD) and quantitation (LOQ) of analytes in cranberry juice supernatant and precipitate extract and hydrolysate.

	cranberry juice supernatant		methanol extract and hydrolysate of juice precipitate	
compound	LOD (µM)	LOQ (µM)	LOD (µM)	LOQ (µM)
gallic acid	0.054	0.16	0.10	0.32
protocatechuic acid	0.11	0.35	0.15	0.46
vanillic acid	0.057	0.17	0.089	0.27
cyanidin 3-O-galactoside	0.1008	0.31	4.1	13
cyanidin 3-O-glucoside	0.055	0.17	3.4	10
cyanidin 3-O-arabinoside	0.047	0.14	0.478	1.4
peonidin 3-O-galactoside	0.059	0.18	0.18	0.53
peonidin 3-O-glucoside	0.060	0.18	0.73	2.2
peonidin 3-O-arabinoside	0.051	0.16	0.91	2.8
cyanidin			4.1	12
peonidin			0.17	0.51

Supplemental Table S2. Quantitated lower limits of detection (LOD) and quantitation (LOQ) of analytes in isolated cranberry juice anthocyanins, extract of precipitate of isolated anthocyanins, and hydrolysate of precipitate of isolated anthocyanins.

	isolated anthocyanins and methanol extract of precipitate of isolated anthocyanins		hydrolysate of precipitate of isolated anthocyanins	
compound	LOD (µM)	LOQ (µM)	LOD (µM)	LOQ (µM)
gallic acid	0.078	0.24	0.13	0.40
protocatechuic acid	0.13	0.39	0.86	2.6
vanillic acid	0.11	0.33	0.22	0.68
cyanidin 3-O-galactoside	1.9	5.6	0.42	1.3
cyanidin 3-O-glucoside	2.2	6.6	0.38	1.1
cyanidin 3-O-arabinoside	0.79	2.4	0.54	1.6
peonidin 3-O-galactoside	1.1	3.3	0.71	2.1
peonidin 3-O-glucoside	1.4	4.1	0.67	2.0
peonidin 3-O-arabinoside	1.4	4.3	1.0	3.1
cyanidin			0.44	1.3
peonidin			0.23	0.70

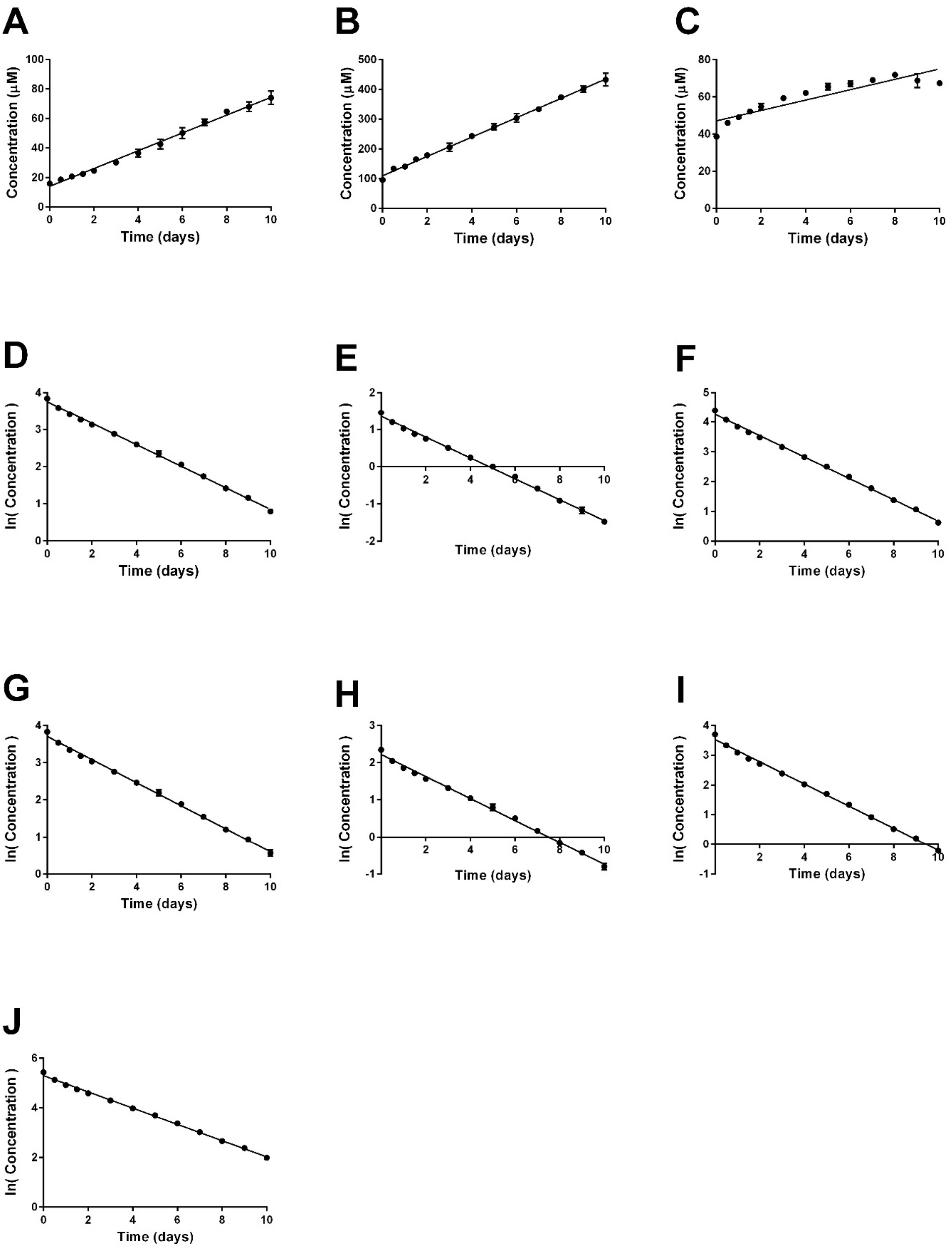

Supplemental Figure S1. Concentration of (A) gallic acid, (B) protocatechuic acid, (C) vanillic acid; natural logarithm of concentration of (D) cyanidin 3-O-galactoside, (E) cyanidin 3-O-glucoside, (F) cyanidin 3-O-arabinoside, (G) peonidin 3-O-galactoside, (H) peonidin 3-O-glucoside, (I) peonidin 3-O-

arabinoside; and (J) the natural logarithm of the sum of concentration of the six aforementioned anthocyanins in cranberry juice supernatant as measured by HPLC during accelerated aging at 50 ºC. All are shown with modeled linear least square regressions for pseudo-zeroth or -first order kinetics: gallic acid, Y = (6.0 ± 0.1) X + (14.0 ± 0.6) (R^2 = 0.9838, non-zero slope $p < 0.0001$); protocatechuic acid, Y = (32.5 ± 0.5) X + (110. ± 2) (R^2 = 0.9905, non-zero slope $p < 0.0001$); vanillic acid, Y = (2.8 ± 0.2) X + (47 ± 1) (R^2 = 0.8101, non-zero slope $p < 0.0001$); cyanidin 3-O-galactoside, Y = (-0.290 ± 0.002) X + (3.75 ± 0.01) (R^2 = 0.9965, non-zero slope $p < 0.0001$); cyanidin 3-O-glucoside, Y = (-0.281 ± 0.002) X + (1.36 ± 0.01) (R^2 = 0.9963, non-zero slope $p < 0.0001$); cyanidin 3-O-arabinoside, Y = (-0.359 ± 0.003) X + (4.26 ± 0.02) (R^2 = 0.9965, non-zero slope $p < 0.0001$); peonidin 3-O-galactoside, Y = (-0.311 ± 0.003) X + (3.71 ± 0.01) (R^2 = 0.9961, non-zero slope $p < 0.0001$); peonidin 3-O-glucoside, Y = (-0.296 ± 0.003) X + (2.22 ± 0.02) (R^2 = 0.9948, non-zero slope $p < 0.0001$); peonidin 3-O-arabinoside, Y = (-0.373 ± 0.003) X + (3.53 ± 0.02) (R^2 = 0.9958, non-zero slope $p < 0.0001$); and sum of anthocyanins by HPLC, Y = (-0.327 ± 0.003) X + (5.30 ± 0.02) (R^2 = 0.9962, non-zero slope $p < 0.0001$).

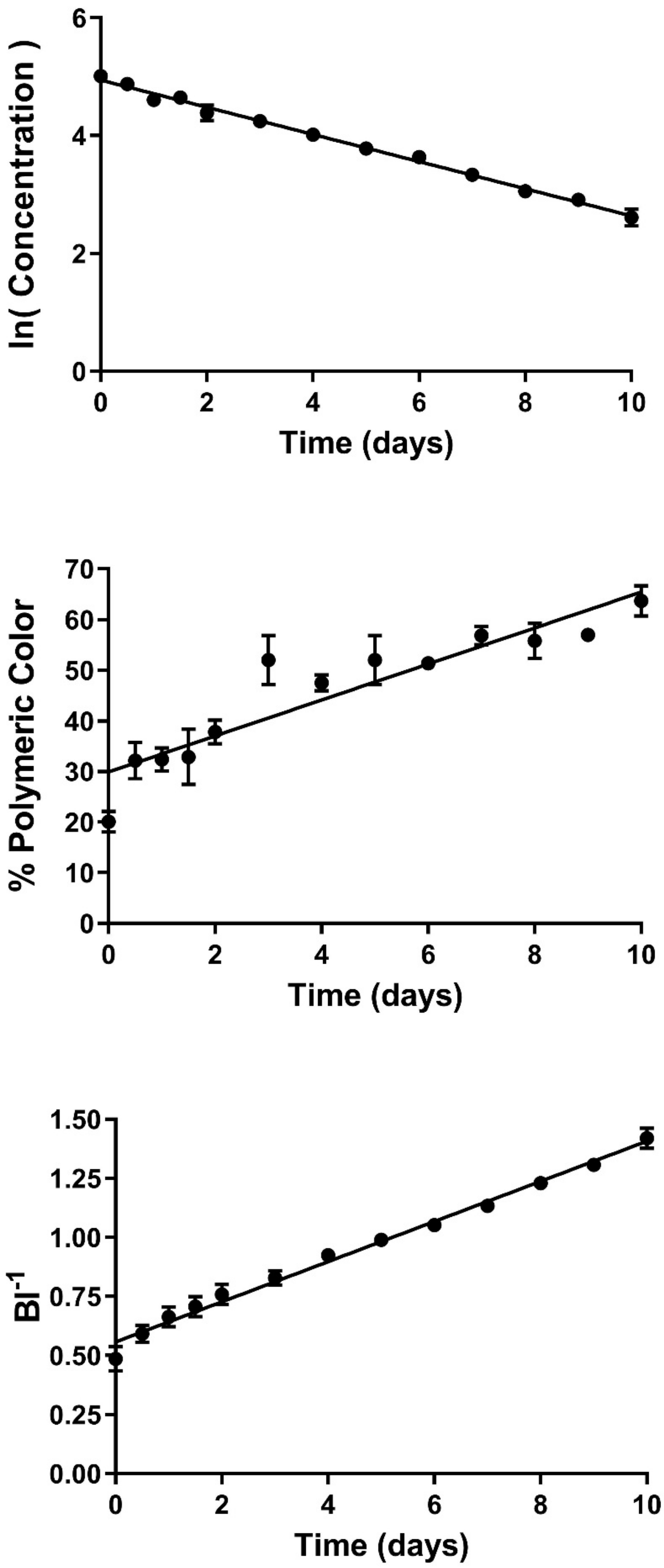
ln(Concentration)
Time (days)
% Polymeric Color
Time (days)
BI-1
Time (days)

Supplemental Figure S2. (A) Natural logarithm of total anthocyanin content (MA) as measured by the pH differential method, (B) percent polymeric color (%PC), and (C) the inverse of browning index (BI) of cranberry juice supernatant during accelerated aging at 50 ºC. All are shown with modeled linear least square regressions for pseudo-zeroth, -first, or -second order kinetics: MA, Y = (-0.231 ± 0.004) X + (4.94 ± 0.02) (R^2 = 0.9842, non-zero slope $p < 0.0001$); %PC, Y = (3.5 ± 0.2) X + (30. ± 1) (R^2 = 0.8102, non-zero slope $p < 0.0001$); and BI, Y = (0.085 ± 0.002) X + (0.557 ± 0.009) (R^2 = 0.9803, non-zero slope $p < 0.0001$).

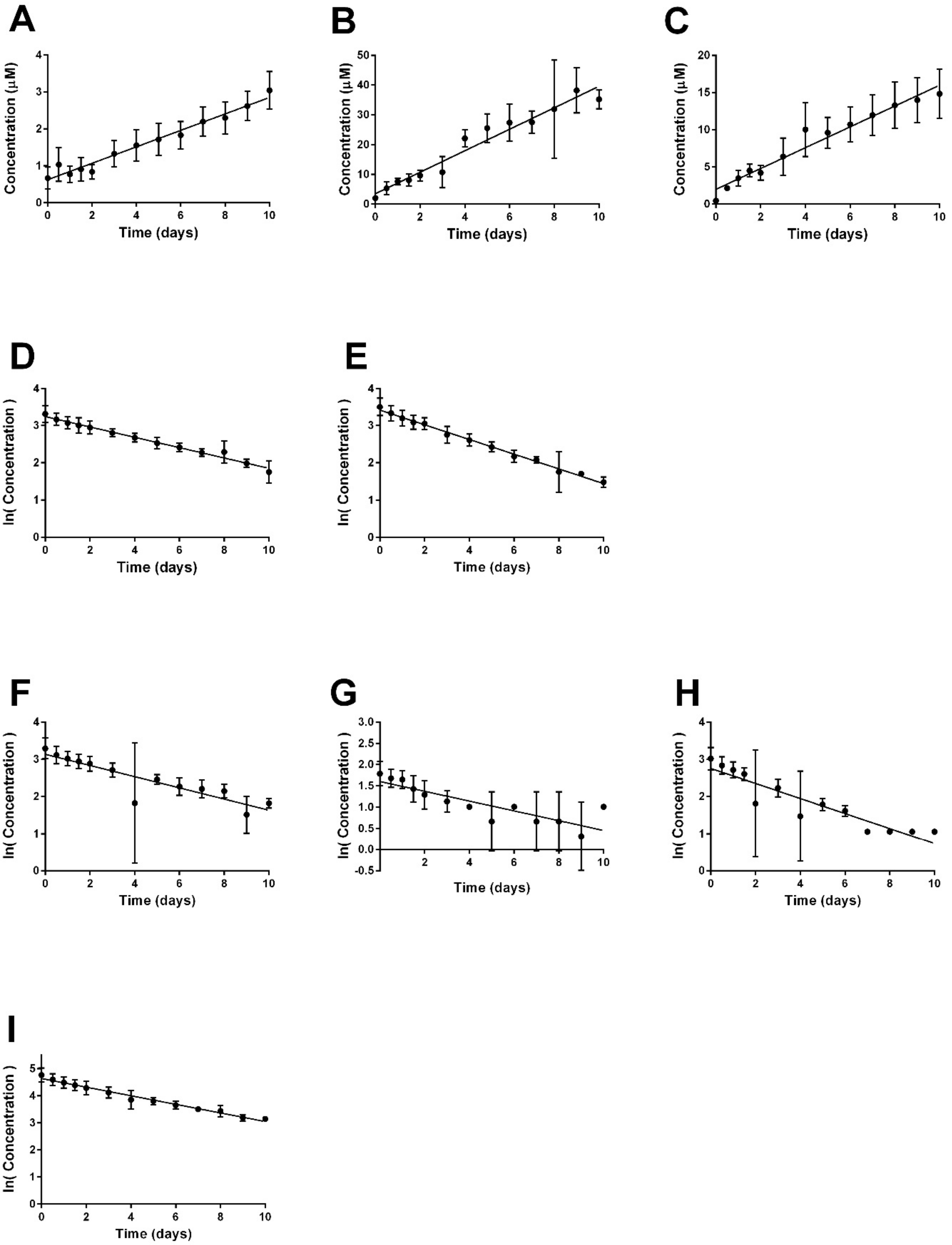

Supplemental Figure S3. Concentration of (A) gallic acid, (B) protocatechuic acid, (C) vanillic acid; natural logarithm of concentration of (D) cyanidin 3-O-galactoside, (E) cyanidin 3-O-arabinoside, (F) peonidin 3-O-galactoside, (G) peonidin 3-O-glucoside, (H) peonidin 3-O-arabinoside; and (I) the natural

logarithm of the sum of concentration cyanidin and peonidin 3-O-galactoside, -glucoside, and -arabinoside of isolated cranberry juice anthocyanins as measured by HPLC during accelerated aging at 50 ºC. All are shown with modeled linear least square regressions for pseudo-zeroth or -first order kinetics: gallic acid, Y = (0.22 ± 0.02) X + (0.62 ± 0.08) (R^2 = 0.8070, non-zero slope $p < 0.0001$); protocatechuic acid, Y = (3.6 ± 0.3) X + (3 ± 1) R^2 = 0.8058, non-zero slope $p < 0.0001$); vanillic acid, Y = (1.4 ± 0.1) X + (2.0 ± 0.5) (R^2 = 0.8091, non-zero slope $p < 0.0001$); cyanidin 3-O-galactoside, Y = (-0.139 ± 0.007) X + (3.24 ± 0.04) (R^2 = 0.8747, non-zero slope $p < 0.0001$); cyanidin 3-O-arabinoside, Y = (-0.197 ± 0.009) X + (3.42 ± 0.05) (R^2 = 0.9104, non-zero slope $p < 0.0001$); peonidin 3-O-galactoside, Y = (-0.15 ± 0.02) X + (3.1 ± 0.1) (R^2 = 0.4920, non-zero slope $p < 0.0001$); peonidin 3-O-glucoside, Y = (-0.12 ± 0.02) X + (1.6 ± 0.1) (R^2 = 0.4151, non-zero slope $p < 0.0001$); peonidin 3-O-arabinoside, Y = (-0.20 ± 0.02) X + (2.8 ± 0.1) (R^2 = 0.5978, non-zero slope $p < 0.0001$); and sum of anthocyanins by HPLC, Y = (-0.159 ± 0.008) X + (4.63 ± 0.04) (R^2 = 0.8841, non-zero slope $p < 0.0001$).

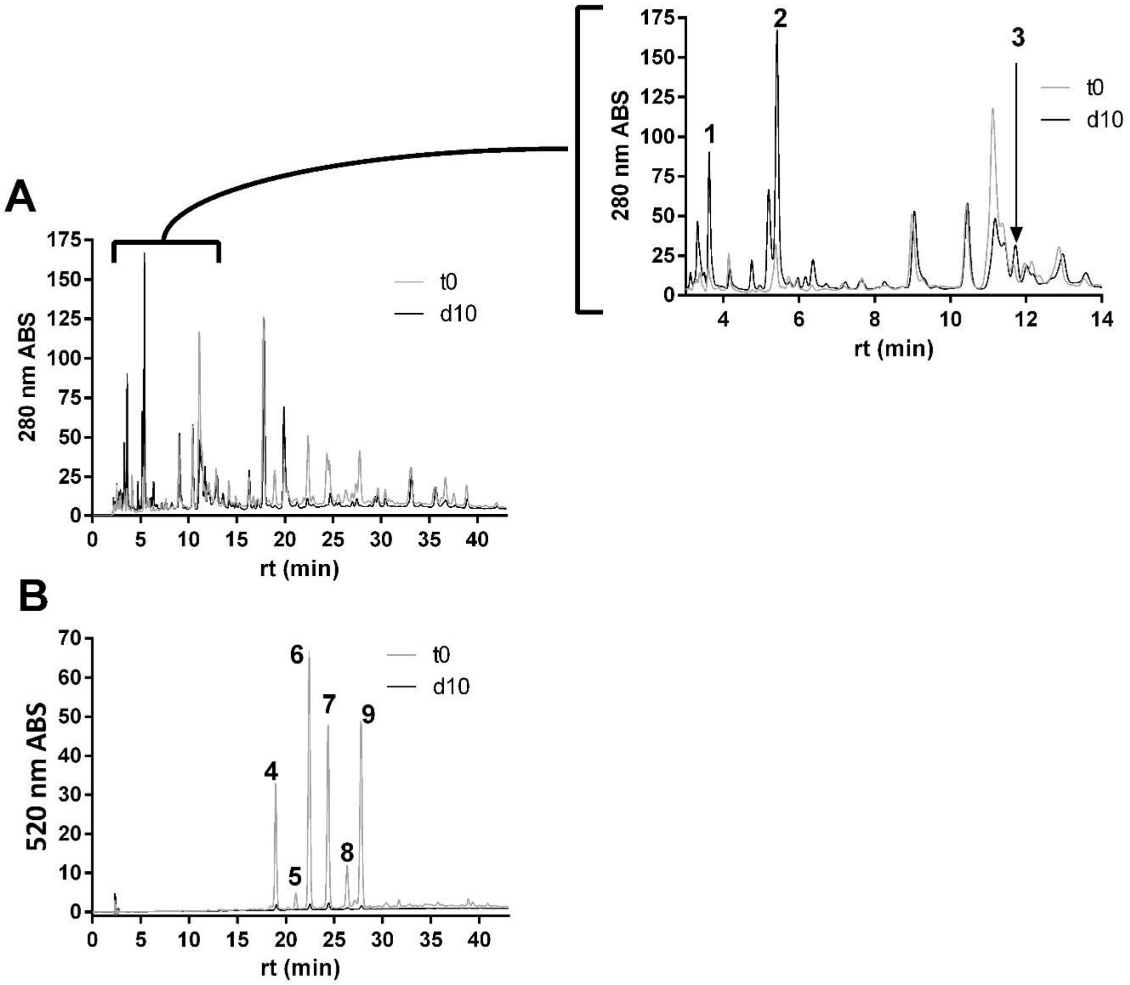

Supplemental Figure S4. Example RP-HPLC chromatograms of freshly prepared juice supernatant (t0) and juice supernatant after 10 days at 50 ºC (d10) (A) at 280 nm and (B) 520 nm. Analytes corresponding to numbered peaks are identified in **Table S3**.

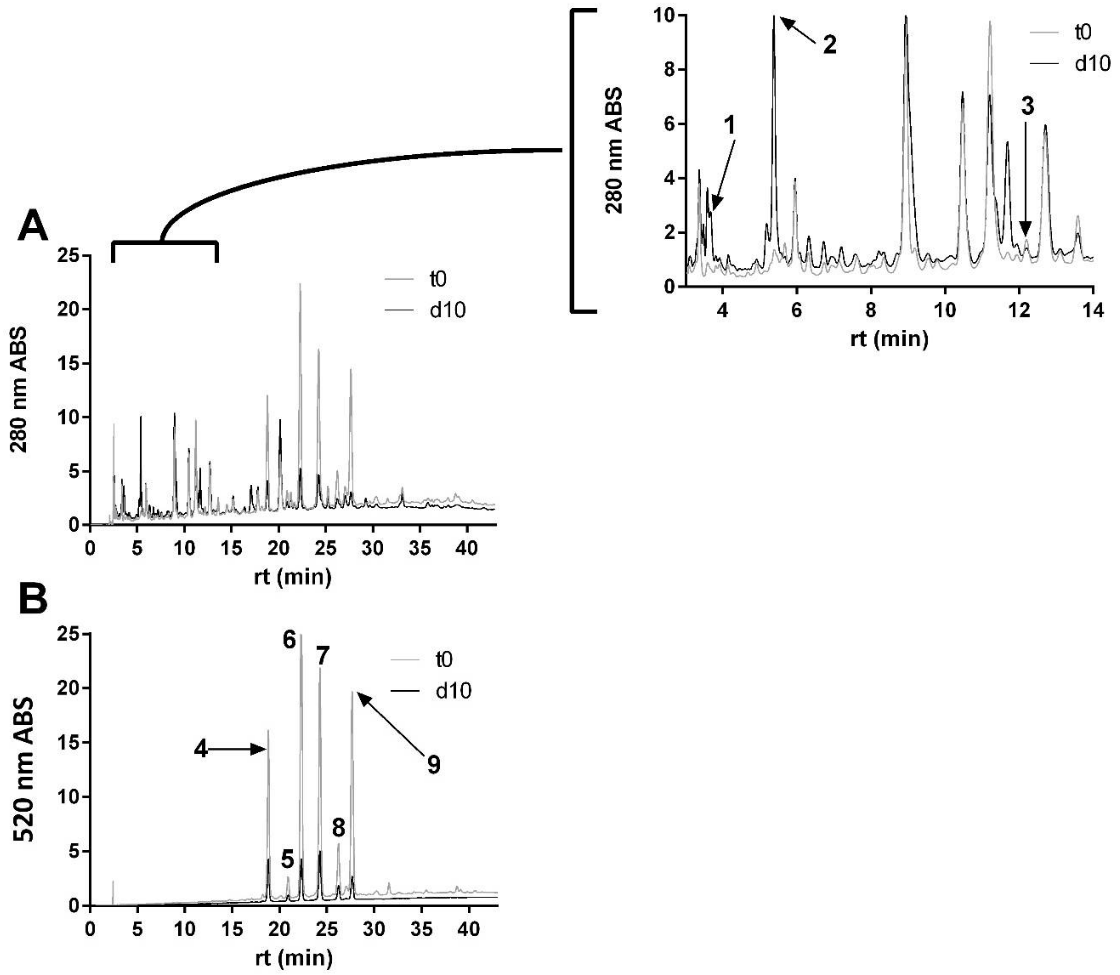

Supplemental Figure S5. Example RP-HPLC chromatograms of isolated cranberry juice anthocyanins (t0) and isolated cranberry juice anthocyanins after 10 days at 50 ºC (d10) (A) at 280 nm and (B) 520 nm. Analytes corresponding to numbered peaks are identified in **Table S3**.

Supplemental Table S3. Peak numbers in example chromatograms and retention time of analytes in HPLC analyses

peak	compound	retention time (min)
1	gallic acid	3.64
2	protocatechuic acid	5.49
3	vanillic acid	11.99
4	cyanidin 3-O-galactoside	19.18
5	cyanidin 3-O-glucoside	21.25
6	cyanidin 3-O-arabinoside	22.67
7	peonidin 3-O-galactoside	24.62
8	peonidin 3-O-glucoside	26.60
9	peonidin 3-O-arabinoside	28.04
10	cyanidin	32.63
11	*tert*-butylhydroquinone	34.05
12	peonidin	39.76

Supplemental Table S4. Percent of each anthocyanin or class of anthocyanins relative to total measured anthocyanin concentration in cranberry juice supernatant and in soluble isolated cranberry juice anthocyanins.

anthocyanin(s)	percent of anthocyanin(s) in cranberry juice supernatant	percent of anthocyanin(s) in isolated cranberry juice anthocyanins[a]	adjusted P values[b]
cyanidin 3-O-galactoside*	20.6 ± 0.6%	23 ± 1%	0.036883
cyanidin 3-O-glucoside	2.39 ± 0.04%	< 5.5%	0.474760
cyanidin 3-O-arabinoside*	35.1 ± 0.3%	28.4 ± 0.5%	0.000028
peonidin 3-O-galactoside*	18.2 ± 0.2%	22.9 ± 0.7%	0.005061
peonidin 3-O-glucoside	5.583 ± 0.007%	5.1 ± 0.3%	0.177654
peonidin 3-O-arabinoside	18.1 ± 0.1%	17 ± 1%	0.467051
cyanidin-glycosides (sum)	58.2 ± 0.3%	55 ± 2%	0.170502
peonidin-glycosides (sum)	41.8 ± 0.3%	45 ± 2%	0.170502

Data are mean ± standard deviation of n = 4 experiments.

[a]For anthocyanins where the concentration was below the quantification limits, values are reported as < LOQ/sum of anthocyanin concentration. Detection and quantification limits are included in **Tables S1** and **S2**.

[b]Percent compositions of anthocyanins of cranberry juice supernatant isolated cranberry juice anthocyanins were compared using an unpaired t test with Welch's correction and Holm-Sidak method to account for multiple comparisons. Reported P values were adjusted to account for multiple corrections. The three anthocyanins indicated (*) had statistically different percent composition in the cranberry juice supernatant and in the isolated cranberry juice anthocyanins.

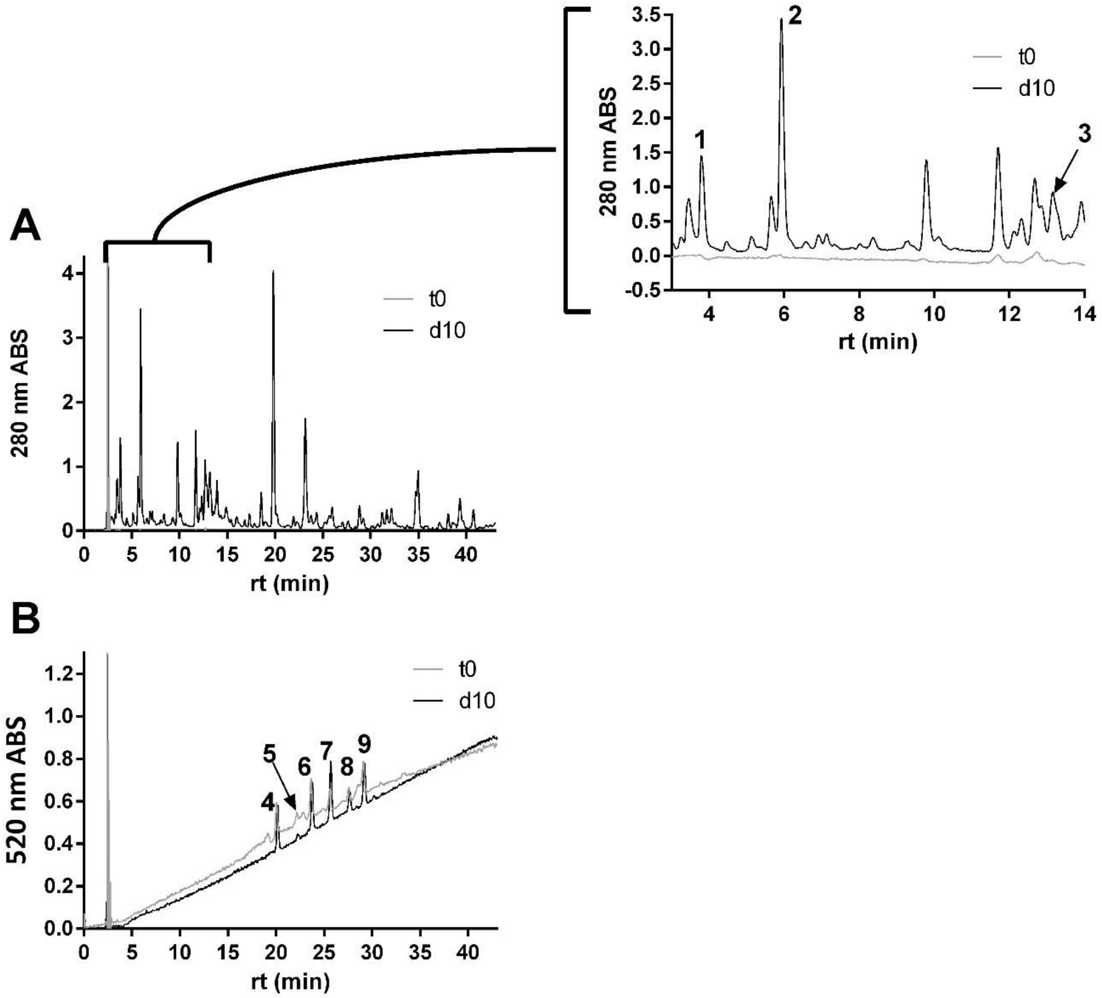

Supplemental Figure S6. Example RP-HPLC chromatograms of methanol extract of precipitate from freshly prepared cranberry juice (t0) and methanol extract of precipitate from cranberry juice after 10 days at 50 ºC (d10) (A) at 280 nm and (B) 520 nm. Analytes corresponding to numbered peaks are identified in **Table S3**.

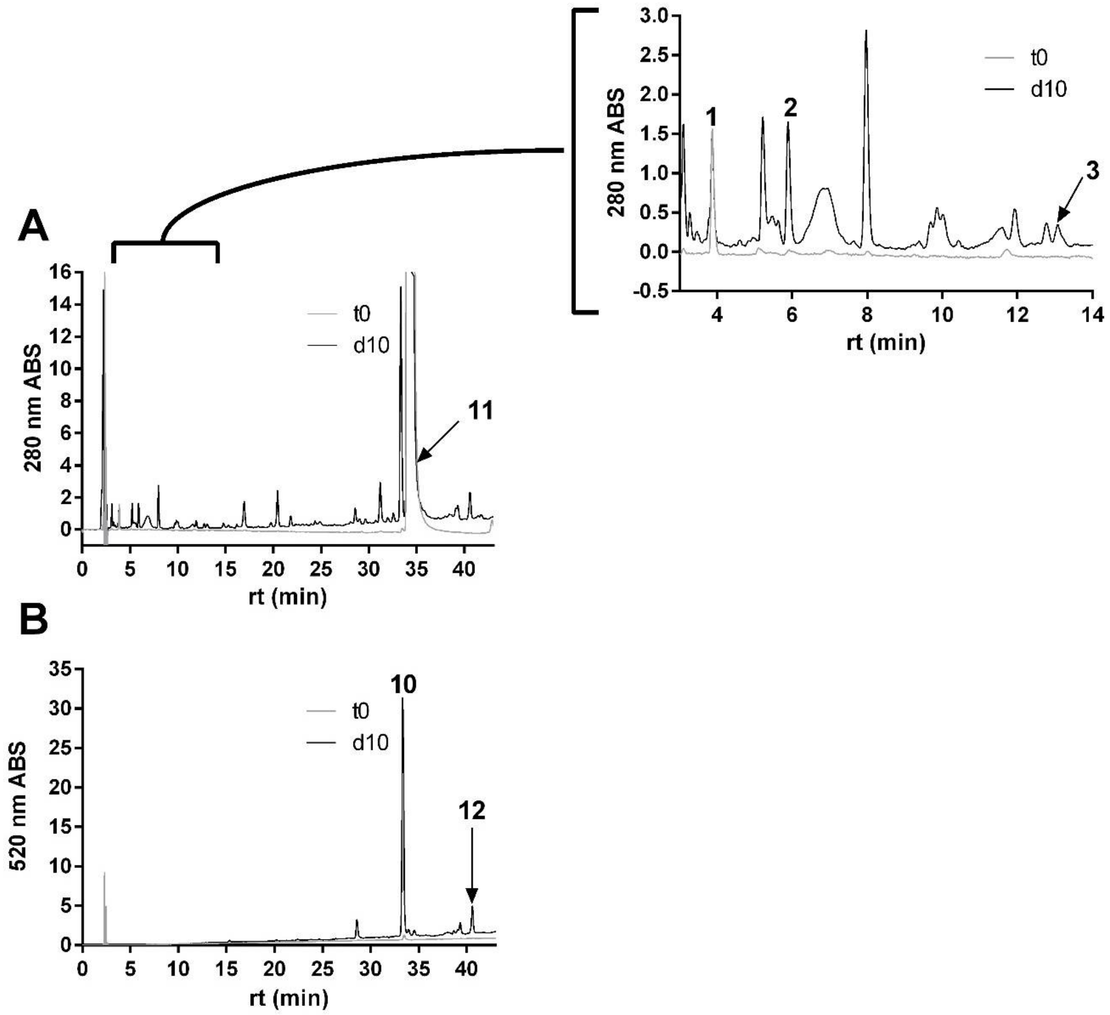

Supplemental Figure S7. Example RP-HPLC chromatograms of hydrolysate of precipitate from freshly prepared cranberry juice (t0) and hydrolysate of precipitate from cranberry juice after 10 days at 50 °C (d10) (A) at 280 nm and (B) 520 nm. Analytes corresponding to numbered peaks are identified in **Table S3**.

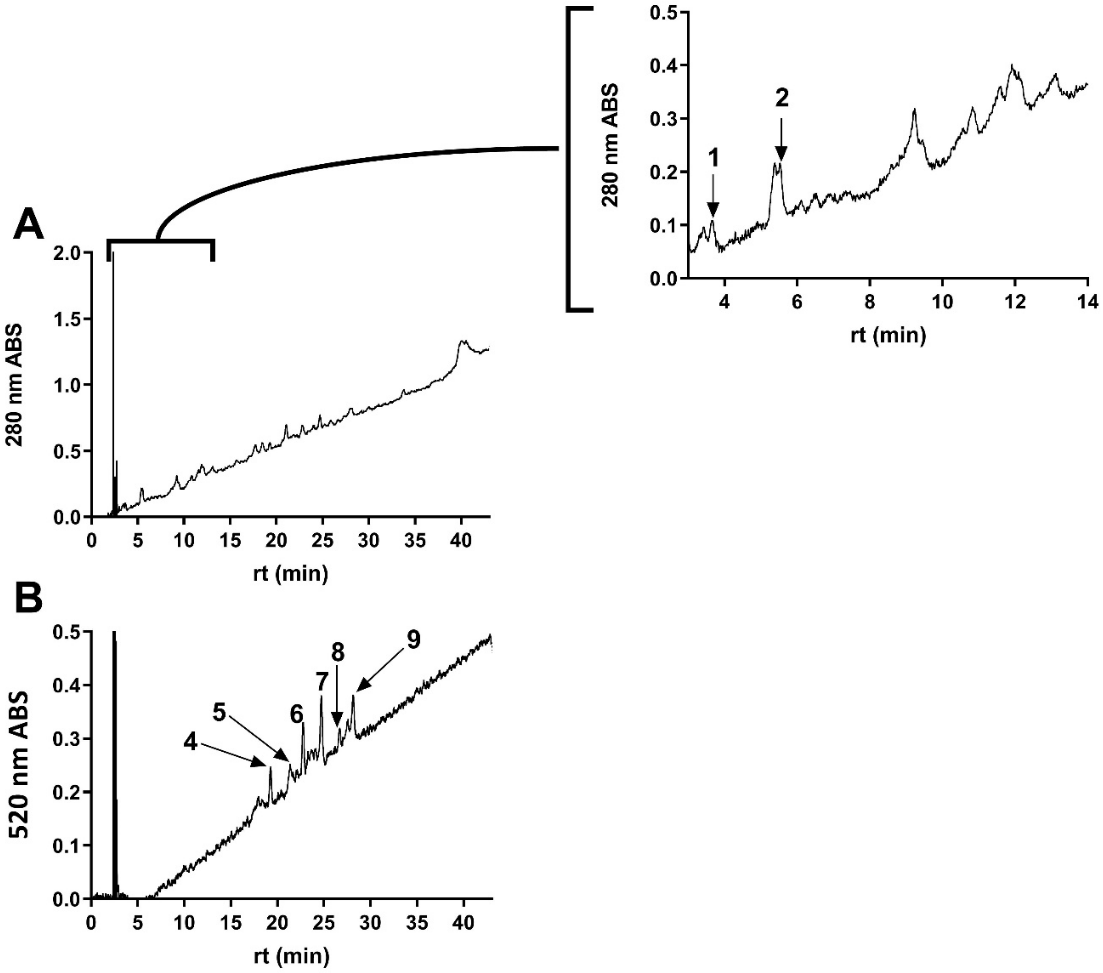

Supplemental Figure S8. Example RP-HPLC chromatograms of methanol extract of precipitate from isolated cranberry juice anthocyanins after 10 days at 50 ºC (A) at 280 nm and (B) 520 nm. Analytes corresponding to numbered peaks are identified in **Table S3**.

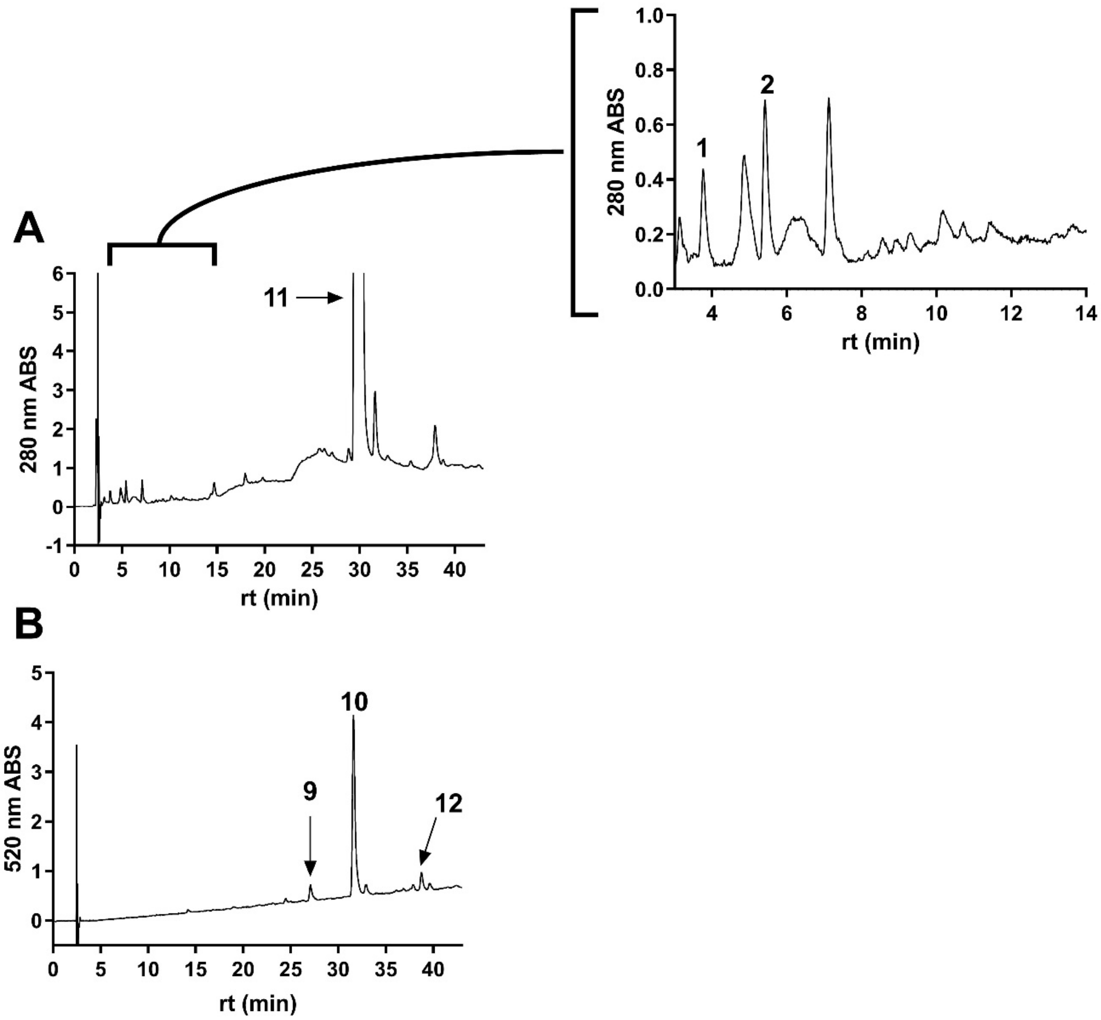

Supplemental Figure S9. Example RP-HPLC chromatograms of hydrolysate of precipitate from isolated cranberry juice anthocyanins after 10 days at 50 ºC (A) at 280 nm and (B) 520 nm. Analytes corresponding to numbered peaks are identified in **Table S3**.

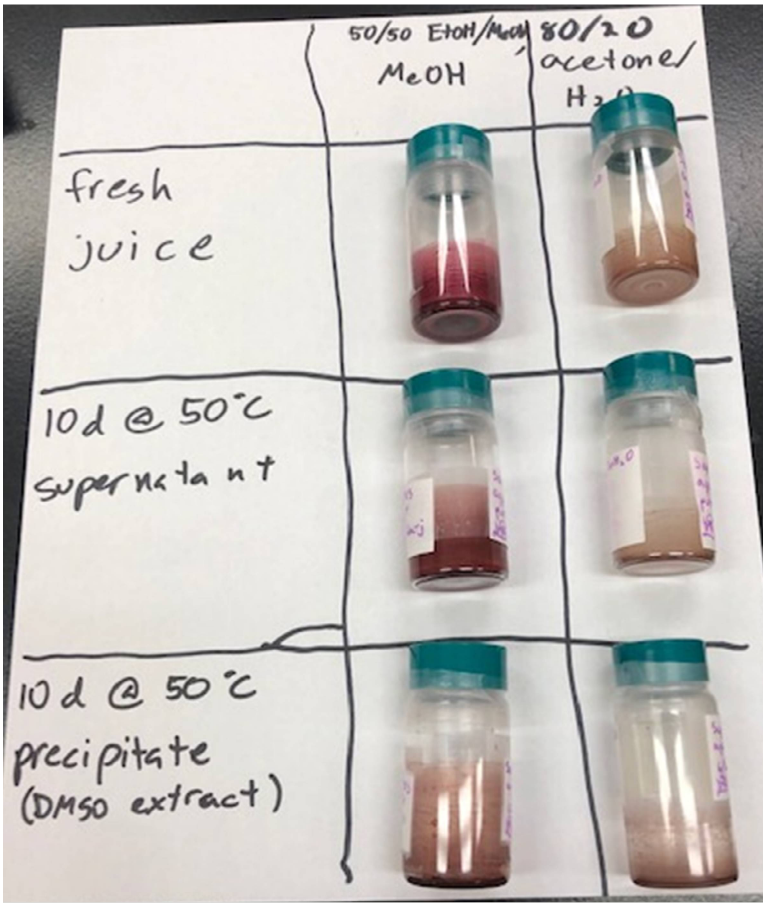

Supplemental Figure S10. Photograph of pigmented samples analyzed via MALDI-TOF.

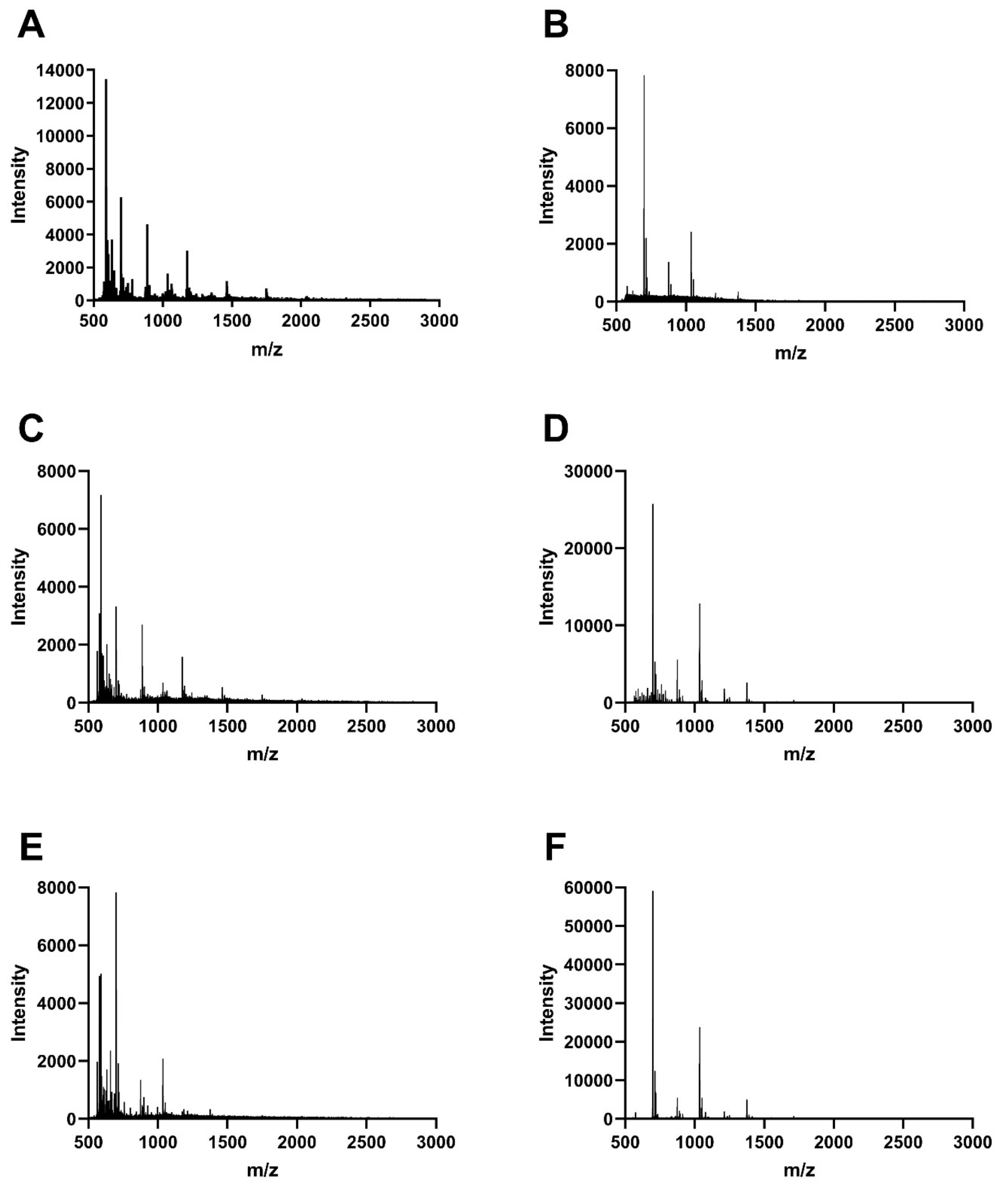

Supplemental Figure S11. MALDI-TOF mass spectra of freshly prepared juice samples and samples of juice after 10 days accelerated aging at 50 ºC all purified by Sephadex LH-20 chromatography. (A) Freshly prepared juice, combined eluates of 50/50 ethanol/methanol and methanol. (B) Freshly prepared juice, eluate of 80/20 acetone/water. (C) Supernatant of juice after accelerated aging, combined eluates of 50/50 ethanol/methanol and methanol. (D) Supernatant of juice after accelerated aging, eluate of 80/20 acetone/water. (E) Precipitate of juice after accelerated aging, combined eluates of 50/50

ethanol/methanol and methanol. (F) Precipitate of juice after accelerated aging, eluate of 80/20 acetone/water.

Supplemental Table S5. Analytical parameters of MALDI-TOF peaks identified as anthocyanin-procyanidins of cranberry juice, freshly prepared or after 10 days accelerated aging at 50 ºC.

Sephadex fraction	anthocyanin	# (epi)catechin units	#A type linkages	anthocyanin-procyandin connection	molecular formula	calculated mass	measured mass	intensity
				**unaged whole juice**				
alcohols	cyanidin-hexoside	1	0	ethylene cross-linked	C38H37O17	765.2025	765.197	347
		2	1	directly bonded	C51H43O23	1023.219	1023.2165	324
		2	1	ethylene cross-linked	C53H47O23	1051.2503	1051.222	411
		3	1	ethylene cross-linked	C68H59O29	1339.3137	1339.2453	193
		3	2	directly bonded	C66H53O29	1309.2667	1309.2329	183
		3	2	ethylene cross-linked	C68H57O29	1337.298	1337.2548	261
		4	2	ethylene cross-linked	C83H69O35	1625.3614	1625.2645	125
	cyanidin-pentoside	1	0	ethylene cross-linked	C37H35O16	735.192	735.1898	645
		2	0	ethylene cross-linked	C52H47O22	1023.2553	1023.2165	324
		2	1	ethylene cross-linked	C52H45O22	1021.2397	1021.2111	405
		3	1	ethylene cross-linked	C67H57O28	1309.3031	1309.2329	183
	peonidin-hexoside	1	0	directly bonded	C37H35O17	751.1869	751.1907	204
		1	0	ethylene cross-linked	C39H39O17	779.2182	779.2144	1019
		2	0	directly bonded	C52H47O23	1039.2503	1039.1715	232
		2	0	ethylene cross-linked	C54H51O23	1067.2816	1067.2345	415
		2	1	directly bonded	C52H45O23	1037.2346	1037.0543	1200
		2	1	directly bonded	C52H45O23	1037.2346	1037.0543	1200
		2	1	ethylene cross-linked	C54H49O23	1065.2659	1065.226	762
		2	1	ethylene cross-linked	C54H49O23	1065.2659	1065.226	762
		3	0	ethylene cross-linked	C69H63O29	1355.345	1355.2495	182
		3	1	ethylene cross-linked	C69H61O29	1353.3293	1353.2463	359
		3	1	ethylene cross-linked	C69H61O29	1353.3293	1353.2463	359
		3	2	directly bonded	C67H55O29	1323.2824	1323.2432	190
		3	2	ethylene cross-linked	C69H59O29	1351.3137	1351.254	259
		4	1	ethylene cross-linked	C84H73O35	1641.3927	1641.2569	141
		4	1	ethylene cross-linked	C84H73O35	1641.3927	1641.2569	141
		4	2	ethylene cross-linked	C84H71O35	1639.377	1639.2758	136
	peonidin-pentoside	1	0	ethylene cross-linked	C38H37O16	749.2076	749.207	775
		3	1	ethylene cross-linked	C68H59O28	1323.3187	1323.2432	190

acetone/water	cyanidin-hexoside	2	0	ethylene cross-linked	C53H49O23	1053.266	1053.084	589
	cyanidin-pentoside	1	0	ethylene cross-linked	C37H35O16	735.192	735.2466	285
	peonidin-hexoside	1	0	directly bonded	C37H35O17	751.1869	751.2423	158
		2	1	directly bonded	C52H45O23	1037.235	1037.125	1945
	peonidin-pentoside	2	0	ethylene cross-linked	C53H49O22	1037.271	1037.125	1945
		2	1	ethylene cross-linked	C53H47O22	1035.255	1035.172	219
			**aged juice supernatant**					
alcohols	cyanidin-hexoside	2	1	ethylene cross-linked	C53H47O23	1051.25	1051.266	223
		3	1	ethylene cross-linked	C68H59O29	1339.314	1339.305	136
		3	2	ethylene cross-linked	C68H57O29	1337.298	1337.322	193
	cyanidin-pentoside	2	1	ethylene cross-linked	C52H45O22	1021.24	1021.257	218
	peonidin-hexoside	2	1	directly bonded	C52H45O23	1037.235	1037.126	553
		2	1	ethylene cross-linked	C54H49O23	1065.266	1065.276	311
		3	1	ethylene cross-linked	C69H61O29	1353.329	1353.308	172
		3	2	ethylene cross-linked	C69H59O29	1351.314	1351.294	197
	peonidin-pentoside	2	0	ethylene cross-linked	C53H49O22	1037.271	1037.126	553
		2	1	ethylene cross-linked	C53H47O22	1035.255	1035.256	249
acetone/water	cyanidin-hexoside	2	0	ethylene cross-linked	C53H49O23	1053.2659	1053.1082	2332
	cyanidin-pentoside	1	0	ethylene cross-linked	C37H35O16	735.192	735.3745	551
	peonidin-hexoside	2	0	ethylene cross-linked	C54H51O23	1067.2816	1067.1025	110
		2	1	directly bonded	C52H45O23	1037.2346	1037.1464	10458
	peonidin-pentoside	2	0	ethylene cross-linked	C53H49O22	1037.271	1037.1464	10458
		2	1	ethylene cross-linked	C53H47O22	1035.2553	1035.146	1259
			**aged juice precipitate**					
alcohols	cyanidin-hexoside	2	0	ethylene cross-linked	C53H49O23	1053.2659	1053.0703	436
	peonidin-hexoside	2	1	directly bonded	C52H45O23	1037.2346	1037.1024	1686
	peonidin-pentoside	2	0	ethylene cross-linked	C53H49O22	1037.271	1037.1024	1686
		2	1	ethylene cross-linked	C53H47O22	1035.2553	1035.1761	123
acetone/water	cyanidin-hexoside	2	1	ethylene cross-linked	C53H47O23	1051.2503	1051.0608	618
	cyanidin-pentoside	1	0	ethylene cross-linked	C37H35O16	735.192	735.2648	1256
	peonidin-hexoside	2	1	directly bonded	C52H45O23	1037.2346	1037.0961	20547
	peonidin-pentoside	2	0	ethylene cross-linked	C53H49O22	1037.271	1037.0961	20547
		2	1	ethylene cross-linked	C53H47O22	1035.2553	1035.0979	2663

Supplemental Table S6. Intensities by constituent anthocyanidin, constituent glycoside, or constituent anthocyanin-proanthocyanidin linkage as percent of total intensity of tentatively identified anthocyanin-proanthocyanidin MALDI-TOF peaks within each analyzed Sephadex fraction

juice fraction	Sephadex fraction	percent of anthocyanin-proanthocyanidins with constituent cyanidin/peonidin	percent of anthocyanin-proanthocyanidins with constituent pentoside/hexoside	percent of anthocyanin-proanthocyanidins with/without ethylene bridge between anthocyanin and proanthocyanidin
unaged whole juice	alcohols	28/72	21/79	70/30
	acetone/water	17/83	48/52	59/41
aged juice supernatant	alcohols	27/73	36/64	80/20
	acetone/water	11/89	49/51	58/42
aged juice precipitate	alcohols	11/89	46/54	57/43
	acetone/water	4/96	56/44	57/43
